# Neural Processing Mechanism of Mental Calculation Based on Cerebral Oscillatory Changes: A Comparison Between Abacus Experts and Novices

**DOI:** 10.3389/fnhum.2020.00137

**Published:** 2020-04-15

**Authors:** Abdelkader Nasreddine Belkacem, Kanako Kiso, Etsuko Uokawa, Tetsu Goto, Shiro Yorifuji, Masayuki Hirata

**Affiliations:** ^1^Department of Computer and Network Engineering, College of Information Technology, United Arab Emirates University, Al Ain, United Arab Emirates; ^2^Department of Neurological Diagnosis and Restoration, Osaka University Graduate School of Medicine, Suita, Japan; ^3^Department of Neurosurgery, Osaka University Graduate School of Medicine, Suita, Japan; ^4^Endowed Research Department of Clinical Neuroengineering, Global Center for Medical Engineering and Informatics, Osaka University, Suita, Japan; ^5^Center for Information and Neural Networks (CiNet), National Institute of Information and Communications Technology, Osaka University, Suita, Japan

**Keywords:** abacus mental calculation, cerebral oscillatory, brain hemispheres activation, neural mechanism, magnetoencephalography, synthetic aperture magnetometry

## Abstract

**Background**: Abacus experts could mentally calculate fast some mathematical operations using multi-digit numbers. The temporal dynamics of abacus mental calculation are still unknown although some behavioral and neuroimaging studies have suggested a visuospatial and visuomotor neural process during abacus mental calculation. Therefore, this contribution aims to clarify the significant similarities and the differences between experts and novices by investigating calculation-induced neuromagnetic responses based on cerebral oscillatory changes.

**Methods**: Twelve to 13 healthy abacus experts and 17 non-experts participated in two experimental paradigms using non-invasive neuromagnetic measurements. In experiments 1 and 2, the spatial distribution of oscillatory changes presented mental calculations and temporal frequency profiles during addition while examining multiplication tasks. The MEG data were analyzed using synthetic aperture magnetometry (SAM) with an adaptive beamformer to calculate the group average of the spatial distribution of oscillatory changes and their temporal frequency profiles in source-level analyses.

**Results**: Using a group average of the spatial distribution of oscillatory changes, we observed some common brain activities in both right-handed abacus experts and non-experts. In non-experts, we detected the right dorsolateral prefrontal cortex (DLPFC) and bilateral Intraparietal sulcus (IPS); whereas in experts, detected the bilateral parieto-occipital sulcus (POS), right inferior frontal gyrus (IFG), and left sensorimotor areas mainly. Based on the findings generated, we could propose calculation processing models for both abacus experts and non- experts conveniently.

**Conclusion**: The proposed model of calculation processing in abacus experts and novices revealed that the novices could calculate logically depending on numerical processing in the left IPS. In contrast, abacus experts are utilizing spatial processing using a memorized imaginary abacus, which distributed over the bilateral hemispheres in the IFG and sensorimotor areas.

## Introduction

For decades, the abacus-based mental calculation has been a unique Asian culture for a long time for rapid and precise calculations. For instance, old Asian people have relied on physical devices, such as the abacus or *Soroban* in Japanese, to perform complex computations. The experts in the abacus can perform some complex computations mentally with fast speed of response and high accuracy of the answer. However, these neural bases of computation processing are still not precisely known; especially, the neural processing mechanism based on cerebral oscillations (e.g., oscillatory changes in the frequency bands, as alpha (8–13 Hz), beta (13–30 Hz) and gamma (>30 Hz) bands). These oscillatory changes associated with specific functional roles (i.e., cognitive processes) over given brain areas (Dimitriadis et al., 2016). The designation of “Abacus experts” refers to those who have gained an unusual ability to operate an abacus for mathematical operations, as well as calculating mentally with an abacus in mind after almost daily training throughout the years.

Training on the abacus-based mental calculation (AMC) has received much attention in neuroscience communities for some clinical and non-clinical applications (Tanaka et al., 2002; Hanakawa et al., 2003; Chen et al., 2006; Hu et al., 2011; Li et al., 2013; Wang et al., 2013, 2017). Most researchers have been trying to understand how the brain works when someone uses an abacus to gain arithmetic skills (Dehaene et al., 1990, 1999, 2003; Dehaene, 1992, 1996, 1997, 2008; Dehaene and Cohen, 1997). There is psychological evidence that abacus experts utilize a mental image of an abacus to recall and manipulate large numbers in solving calculation problems; however, the neural correlates underlying this expertise is still unknown (Cohen et al., 1997, 2004; Cohen Kadosh et al., 2007, 2008), and the mathematical language has always been compared to natural language (Amalric and Dehaene, 2016, 2019). Usually, if someone asks abacus experts “how they pull through any mental calculation, they all say, “We do the calculation by using an abacus within my brain.”

Former scholarly studies have covered some relevant active brain areas using positron-emission tomography (PET) and functional magnetic resonance imaging (fMRI) for detecting brain activities related to mathematical and calculation processing, while the importance of the parietal lobe and frontal lobe had always been visible (Dehaene, 1996; Cohen et al., 2000; Rickard et al., 2000). However, since the information obtained from PET and fMRI is based mainly on changes in blood flow, metabolism, and secondary to electrophysiological activities, the time resolution is not high, and it is difficult to capture the reaction at the earliest time-based latency. There are also some prior experimental studies (e.g., Pauli et al., 1994; Iguchi and Hashimoto, 2000) used electroencephalogram (EEG) to measure brain electrical activity directly. In contrast, it is still difficult to capture the mental calculation-based reaction time by using the EEG technique due to its low spatial resolution and high-frequency components.

In this research study, we employed magnetoencephalography (MEG) to measure specific brain activity during mental calculation, where its signals considered as cerebral rhythm changes. The aperture synthetic magnetometry (SAM) method using a nonlinear beamforming approach analyzed the generated MEG signals to investigate brain activity during calculation processing (Taniguchi et al., 2000; Robinson et al., 2004). The SAM method is characterized by canceling noise using a spatial filter and having a high spatial resolution. Furthermore, it is possible to obtain the brain activity region associated with each task by setting the region of interest in the cranium to a lattice shape (voxel) and statistically comparing the signal intensities before and after performing the task in each voxel. Some studies based on MEG have been focusing on decoding the processing stage of mental arithmetic calculations at the single-trial level (Pinheiro-Chagas et al., 2018).

We focused the attention on abacus experts who have sound skills to numbers, along with general brain computation processing mechanisms of healthy subjects. For instance, In Japan, the abacus has been staying in use as a calculation device for a long time. When training an abacus for many years, calculation speed would be faster than the untrained person; so, calculation results could be derived with a high correct answer rate. Also, several reports indicate that the ability to manipulate numbers in memory is excellent, and some prior studies were focusing on special computing abilities (e.g., high calculation processing speed) to have concluded that abacus experts are visual-spatial learners (e.g., Tanaka et al., 2002; Hanakawa et al., 2002; Chen et al., 2006; Wu et al., 2009). To the best of our knowledge; however, mental calculation based on oscillatory changes has not been studied thoroughly using magnetoencephalography.

Although it is being clarified that calculations are made to, it has not been thoroughly studied yet using the magnetoencephalography to the best of our knowledge. Therefore, in this study, we have been conducting research aiming to clarify the difference between the computational processing mechanism of the skilled abacus and non-skilled persons using magnetoencephalography. So far, research has been done on the difference in the calculation process when mentalizing the addition, and non-experts sequentially calculate mental arithmetic with numeric morphological recognition, numerical processing, numeric inner words, working memory, calculation execution processing. While processing, abacus experts got the result that they were doing through several processes, including internal language and calculation processing, at the same time.

However, the additional task used in this study was very easy for experts, and the load on the brain might be substantially different for each subject. Therefore, we used multiplication tasks too to select additional and multiplication tasks based on the level of difficulty, which could be calculated within a particular time by carrying out preliminary experiments and used them in this study which makes the burden on the brain for each subject became equal. The primary driver of this research study is not only clarifying the localization analysis by using the SAM method but also the time-frequency analysis and clarifying the processing process in the brain during multiplication and mental arithmetic.

Therefore, we investigated calculation-induced neuromagnetic responses based on cerebral oscillatory changes. These oscillatory changes are now widely used for functional neuroimaging studies. The present study aims to clarify the spatiotemporal distribution of the cerebral oscillatory changes during mental calculations using synthetic aperture magnetometry (Ihara et al., 2003a,b; Hirata et al., 2007, 2010) and to elucidate the processing mechanism of mental calculation to elucidate the difference between abacus experts and non-experts using magnetic source imaging from magnetoencephalography (MEG) signals. Understanding the neural mechanism of abacus might lead to enhance the calculation ability for patients with Acalculia who are unable to perform mathematical calculations, although some alternative numerical processes are still available to them. This article presents the first model based on the temporal frequency profile of oscillatory changes. Two groups of abacus experts and non-experts were asked to perform some mental calculations to analyze the similarities and differences between them by looking to their temporal profiles to design neural processing models for abacus and non-abacus experts then we compared our results to previous studies (Cohen et al., 1997; Dehaene et al., 1999, 2003; Ishii et al., 1999).

## Materials and Methods

### Participants

Healthy volunteers (17 non-experts from 21 to 55 years of age, and 12–13 abacus experts from 18 to 35) participated in this study to investigate calculation-induced neuromagnetic responses based on cerebral oscillatory changes using non-invasive measurement. For mental addition (+) experiments, the age of non-expert participants is from 21 to 55 years old (Average ± Standard deviation: 24.9 ± 7.86) and the age of experts is from 19 to 24 years old (21.9 ± 2.84). Only one non-expert volunteer was middle-aged (55 years old). For multiplication (×) experiments, the age of non-expert participants is from 21 to 55 years old (25.7 ± 9.3) and the age of experts is from 18 to 35 years old (23.5 ± 3.7). However, there was no statistically significant age difference between participants in both mental calculation experiments, which means that there is no age effects. All participants (experts and non-experts) are right-handed. Abacus experts were certified by the authority for their skills, which were ranging from 2 Kyu to 10 Dan according to the Japanese abacus ranking system (i.e., One Dan is one’s degree or level of expertise and knowledge). These experts got abacus training for 6–27 years in their life, and their abacus ranking varies from one person to another.

All participants who were informed in detail about the research purpose and possible consequences of the MEG experiment have signed upon an explicit written consent. The Ethics Committee at Osaka University Hospital approved the conduct of this study, and the experimental protocol was carried out according to the latest version of the Declaration of Helsinki. The T1 structural MRI scans were performed to obtain DICOM images of the head and brain structures in slices for all participants. The acquisition of individual anatomical MRIs of participants was combined with MEG data for getting more precise source localization.

### Experimental Paradigm and Protocol

We performed the MEG recording and MRI at the Osaka University Hospital (Japan). Neuromagnetic brain activity was measured with a 64-channel MEG system equipped with the whole-head array of first-order radial SQUID gradiometers (NeuroSQUID Model 100, CTF Systems Inc., Canada).

We performed two experiments under simple and complex conditions based on the three mental-operation tasks; these were: (i) numeral or calculation; (ii) observation; and (iii) verbal or confirmation (Hanakawa et al., 2002). The participant was in a sitting position, and a projection screen was fixed in front of the eyes. Visual stimuli were shown on the screen with a visual stimulus presentation system (Presentation, Neurobehavioral Systems, Albany, CA, USA) and a projector (12.5 × 16 × 20″) outside the shielded room. In both experiments, one of the mathematical operation types, i.e., addition or multiplication, was presented on the screen in front of the subject. Then, the subject tries to answer the right answer in his/her mind as instructed upon the presentation of the execution cue. Each trial consisted of the following three phases; these were: (i) the instruction/preparation phase; (ii) the execution phase; and (iii) the rest phase.

**Figure 1:**
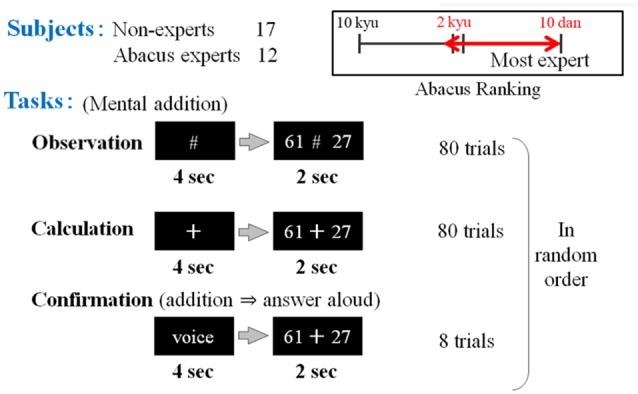
The first experimental paradigm. The participants (17 non-experts and 12 abacus experts) were instructed to perform or confirm at least a mental addition of 2 digits plus 2 digits’ number, immediately after numbers were presented (e.g., 61 + 27). Participants (novices and experts in using a Soroban) were asked to perform three tasks: observation, calculation, and confirmation.

**Figure 2:**
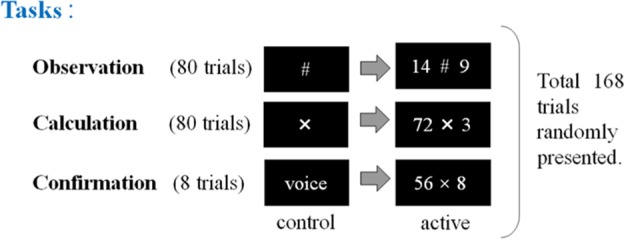
The second experimental paradigm. The participants (17 non-experts and 13 abacus experts) were instructed to perform, observe, or confirm some arithmetic operations by multiplying a two-digit number by a one-digit number (e.g., 72 × 3), immediately after numbers were presented. Participants (novices and experts in using a Soroban) were asked to perform three tasks: calculation, observation, and confirmation. “X” means the execution of mental calculation and “#” means just observation of numbers.

In the first experimental paradigm, the participants (17 non-experts and 12 abacus experts) were instructed to perform at least a mental addition of two digits plus two digits’ number, immediately after numbers were presented. Confirmation task was prepared to confirm whether the subject performs the task adequately or not but this task was excluded from analysis in this study. We prepared two kinds of tasks in the addition experiment. For the first task, the participants were asked to perform the mental calculation. A black fixation cross (visual fixation condition “+”) was presented after each trial for the preparation phase (see Figure 2). This digit stimuli “+” was presented for 4 s on the center of a screen (Figure 1). The participant was instructed to mentally add the presented series of digits without moving their body, especially, fingers. After the presentation of these digit stimuli “+,” series of digits was presented for 2 s. For the second task, the participants were asked to judge whether the addition answer in their mind and the test digit stimuli were the same or different, by answering aloud after each trial. The experimental session consisted of 80 trials for calculation and observation tasks and eight trials for confirmation in a random order.

In the second experimental paradigm, the participants (17 non-experts and 13 abacus experts) were instructed to perform mental multiplication at least by multiplying a two-digit number by a one-digit number (e.g., 72 × 3). We prepared three kinds of tasks in the multiplication experiment (calculation, observation, and confirmation). In the confirmation task, the subjects were asked to speak the answer loudly to confirm whether they perform the given task as requested. This experimental session consisted of 168 trials in total including calculation and confirmation tasks (see Figure 2).

However, we adjusted the task difficulty for both experiments to equalize the task demand for each subject and we used full power metal calculation to detect the calculation-related responses effectively. In each subject, we tested calculation performance (calculation speed and accuracy) in advance before MEG measurements, and equalized task difficulty for each subject. For instance, the subject was instructed to perform 2 × 1 digits, 2 × 2 digits, 3 × 2 digits, 3 × 3 digits, and 4 × 3 digits. Each case included 40 calculations. Then, we recorded accuracies of correct answers and times required for calculation. We found no significant difference between experts and non-experts.

### Data Acquisition and Pre-processing

The neuromagnetic activity was sampled at 1,000 Hz, after which the temporal extension of the signal space separation method (tSSS) was used to suppress noise and artifacts generated by the sensors and sources of interference located very close to the MEG sensors. A band-pass filter from 0.5 to 100 Hz and a 60-Hz notch filter was applied to reduce some high-frequency noises and eliminate AC line noise.

**Figure 3:**
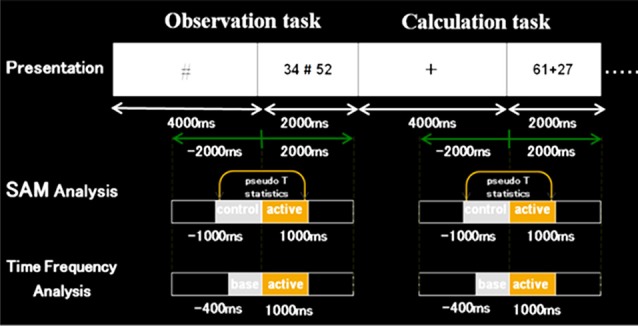
Schematic diagram of the first experimental paradigm. This figure shows a schematic diagram of the task presentation, and a time window of synthetic aperture magnetometry and time-frequency analyses. First, in control stimuli, we presented “+” or VOICE for 4 s, which means what the subject should do in active stimuli. Then, in active stimuli, numbers for calculation were presented for 2 s. In SAM analysis, we compared 1 s before and after the presentation of numbers. Also, regarding time-frequency analysis, the baseline was set for 400 ms before the number presentation.

**Figure 4:**
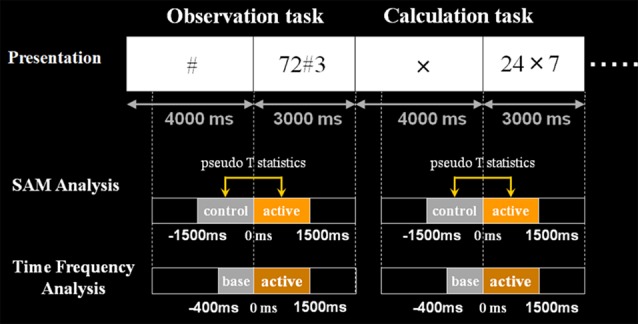
Schematic diagram of the second experimental paradigm. This figure shows the schematic diagram of the task presentation and analyses the time window. First, in control stimuli, we presented X, # or VOICE for 4 s, which means what the subject should do in active stimuli. Then, in active stimuli, numbers for calculation were presented for 3 s. In SAM analysis, we compared 1.5 s before and after the presentation of numbers. Also, regarding time-frequency analysis, the baseline was set for 400 ms before the number presentation. Analyzed frequency bands are as shown here.

For analyses, we used a beamforming method called synthetic aperture magnetometry (SAM) with an adaptive beamformer to obtain high spatial resolution. Besides, we introduced a group analysis to exclude the inter-individual variance. The following Figures 3, 4 show the time windows of the tasks and analyses used in this study. As for SAM analyses, we compared 1 s before and after number presentation. Moreover, concerning time-frequency analyses, the baseline was set from −400 ms to 0 s. Frequency bands were divided into these five bands; ranging from theta to high gamma bands.

We used synthetic aperture magnetometry (SAM) group analysis; SAM was also applied for MEG analysis using an adaptive beamformer. SAM has high spatial resolution using virtual narrow apertures with which we could detect a high-frequency response because this method does not include the averaging process that otherwise cancels out the high-frequency components. SAM group analysis detects common brain activities from individual SAM images using statistical non-parametric mapping. For group analysis, we used statistical non-parametric mapping (SNMP), which is an option of SPM delivered by the Welcome Department of University College London (UCL). We calculated the non-parametric pseudo-t-statistic images based on the variance-covariances of the voxel-level variances for each frequency band, Family-Wise Error rate (FWE) for *p* = 0.001, and MNI coordinates (X, Y, Z) for Brodmann area and the anatomical localization (see Supplementary Tables S1–S8). We also calculated time-frequency analysis using a Morlet wavelet transform to elucidate temporal dynamics of the calculation process using MATLAB 2016a software (Mathworks, Natick, MA, USA).

## Results

### Spatial Distribution of Oscillatory Changes

We analyzed the spatiotemporal frequency patterns of oscillatory changes for experts and non-experts using SAM to understand better source based-brain activation related to abacus experts and investigate calculation-induced neuromagnetic responses based on cerebral oscillatory changes using source level-based MEG signals. The frequency bands are theta (4–8 Hz), alpha (8–13 Hz), beta (13–25 Hz), low gamma (25–50 Hz), high gamma (50–100 Hz). In this study, when subjects performed mental calculations, the magnitude of neuromagnetic fields and the frequency power of brain activities were either increased or decreased in both brain hemispheres; these phenomena are termed as an event-related magnetic field (ERF) for the magnetic fields, event-related synchronization (ERS) and event-related desynchronization (ERD) for the frequency power (Pfurtscheller, 1977, 1992). The spatiotemporal distributions of ERD and ERS could be obtained precisely using SAM. We used this technique to investigate language processing based on cerebral oscillatory changes and have previously reported that cerebral oscillatory changes during silent reading are localized in language-related areas (Hirata et al., 2004, 2007, 2010; Ihara et al., 2003a).

In non-experts, we found: (i) power increase (ERS) in frequency band Theta “θ” in the bilateral frontal pole; (ii) power decrease (ERD) from frequency band Alpha “α” to Beta “β” in the bilateral intraparietal sulcus and the bilateral inferior temporal regions also; (iii) power decrease in low gamma (low γ ERD) in the left inferior and middle frontal gyrus and also in the right dorsolateral prefrontal cortex (DLPFC); and (iv) power increase in high gamma band (high γ ERS) in the bilateral medial occipital regions (for observation and calculation tasks, see Supplementary Figures S1, S2, S13, S14).

**Figure 5:**
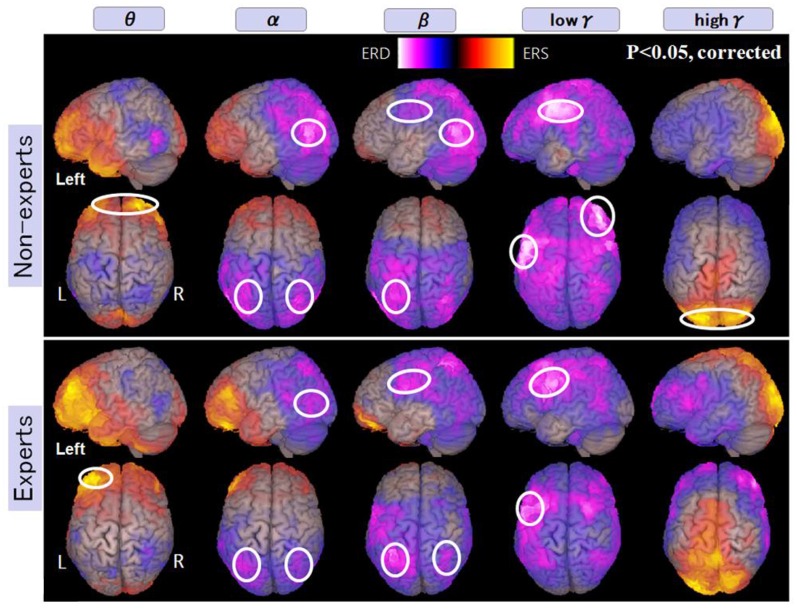
A group average of the spatial distribution of oscillatory changes for the first experiment (mental addition during calculation task). The time window is between −1,000 and 1,000 ms. The frequency interval (all frequency bands) ranges from 4 Hz to 100 Hz. Magenta color shows ERD and orange color shows ERS. The circled areas indicate statistically-significant oscillatory changes. The significant differences observed (*p* < 0.05, corrected) in some brain areas are surrounded by a white circle.

In abacus experts, we found the spatial and frequency distributions are very similar to those of non-experts, but there are two different points in spatial distribution; these were: (i) in non-experts, α ERD was dominant in the temporal region, but in experts, β ERD was more prominent in the parietal region; and (ii) low γ ERD in the right DLPFC was specific to non-experts only. Figure 5 shows the spatial distribution of calculation-induced oscillatory changes revealed by SAM group analyses for both abacus experts and novices. The circled areas in these figures indicate statistically significant oscillatory changes (for observation and calculation tasks, see Supplementary Figures S3, S4, S15, S16).

**Figure 6:**
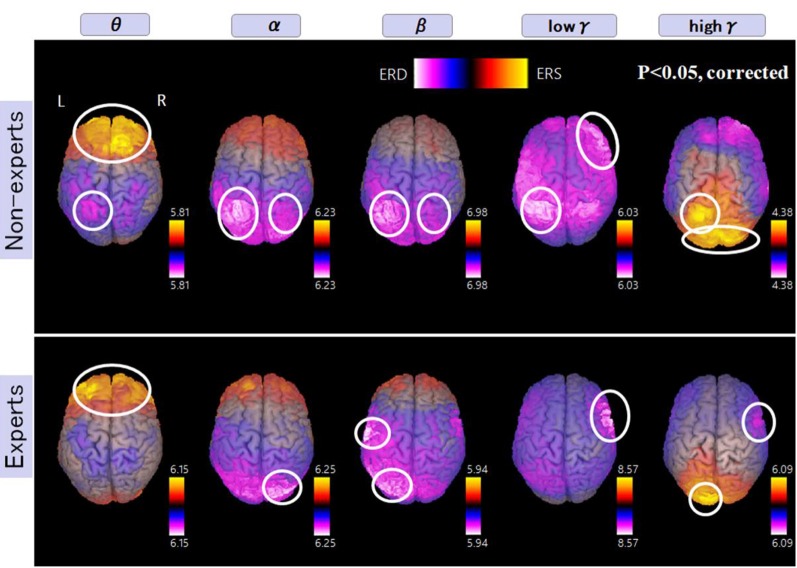
A group average of the spatial distribution of oscillatory changes for the second experiment (mental multiplication during calculation task). The time window is between −1,500 and 1,500 ms. The frequency interval (all frequency bands) ranges from 4 Hz to 100 Hz. Magenta color shows ERD and orange color shows ERS. The circled areas indicate statistically-significant oscillatory changes. The significant differences observed (*p* < 0.05, corrected) in some brain areas are surrounded by a white circle.

Figure 6 shows more activated areas related to mental multiplication tasks. The medial prefrontal cortex and occipital cortex were commonly observed in both experts and non-experts. Specific areas to non-experts are as follows, left dominant parietal mainly intraparietal sulcus (IPS), and right DLPFC. On the other hand, specific areas to abacus experts include bilateral parieto-occipital sulcus (POS), right inferior frontal gyrus (IFG), and left sensorimotor cortex. In the discussion section, we are going to speculate the function of each detected area.

### The Temporal Frequency Profile of Oscillatory Changes

**Figure 7:**
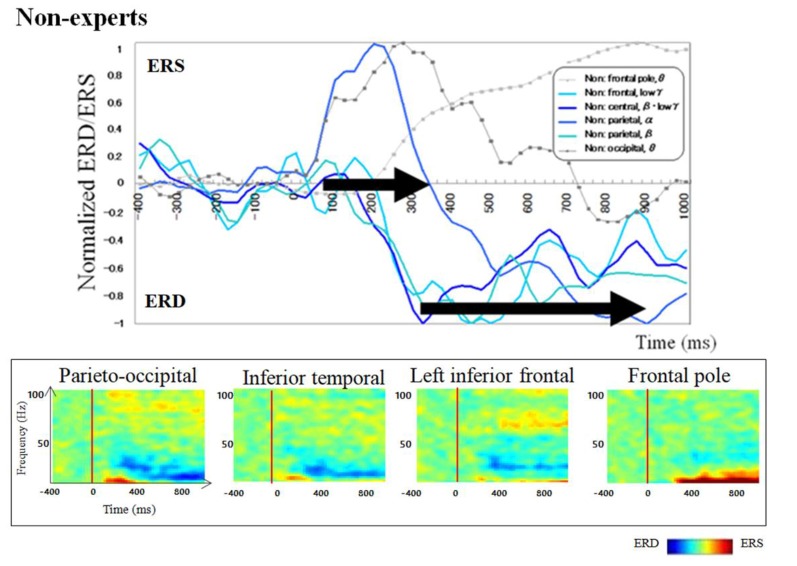
A group average of a time-frequency profile of oscillatory changes for non-experts during mental addition experiments. The upper graph shows the time course of the normalized oscillatory changes in the frequency bands specific to the detected areas and lower graphs show time-frequency spectrograms in each area. The time window is between −400 and 1,000 ms; the movement onset is at 0 ms, and the frequency interval ranges from 4 Hz to 100 Hz. The red areas in the time-frequency maps indicate increases in power (ERS), and the blue areas indicate decreases in power (ERD). In the figure legend, “Non.” means non-abacus experts.

**Figure 8:**
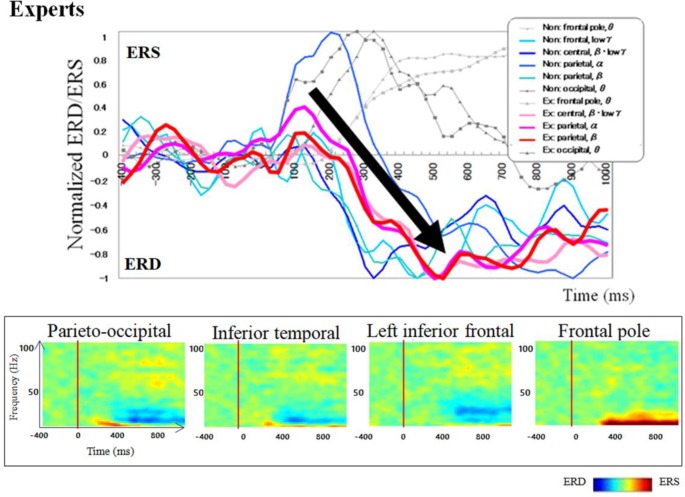
A group average of a time-frequency profile of oscillatory changes for Abacus experts during mental addition experiment. The upper graph shows the time course of the normalized oscillatory changes in the frequency bands specific to the detected areas and lower graphs show time-frequency spectrograms in each area. The time window is between −400 and 1000 ms; the movement onset is at 0 ms, and the frequency interval ranges from 4 Hz to 100 Hz. The red areas in the time-frequency maps indicate increases in power (ERS), and the blue areas indicate decreases in power (ERD). In the figure legend, “Non.” means non-abacus experts and “Ex.” means abacus experts.

Regarding brain activation, we observed some differences in temporal profile for experts and novices using sensor level analysis. We observed, in non-experts, ERDs start serially from parietal, then inferior frontal, dorsolateral prefrontal and finally parietal region again. In contrast, in abacus experts, ERDs start simultaneously in parietal and frontal regions. Figures 7, 8 display group average of temporal profile and time-frequency profile of oscillatory changes during the first experimental paradigm while the upper graph shows the time course of the normalized oscillatory changes in the frequency bands specific to the detected areas and lower graphs show time-frequency spectrograms in each area. For observation and calculation tasks, see the spatiotemporal frequency profile of oscillatory changes in Supplementary Figures S5–S10, S17–S24.

**Figure 9:**
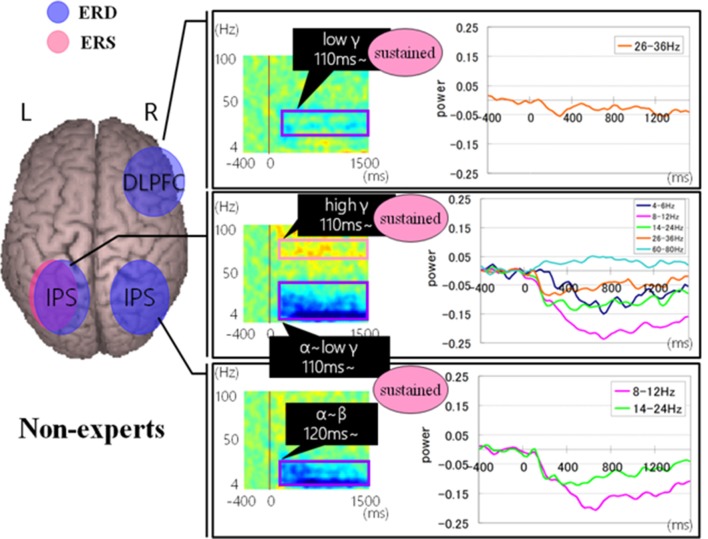
A group average of time-frequency analyses in non-experts during the mental multiplication experiment. The time window is between −400 and 1500 ms; the movement onset is at 0 ms, and the frequency interval ranges from 4 Hz to 100 Hz. The red areas in the time-frequency maps indicate increases in the high gamma power (ERS), and the blue areas indicate decreases in the beta/alpha/low gamma power (ERD).

**Figure 10:**
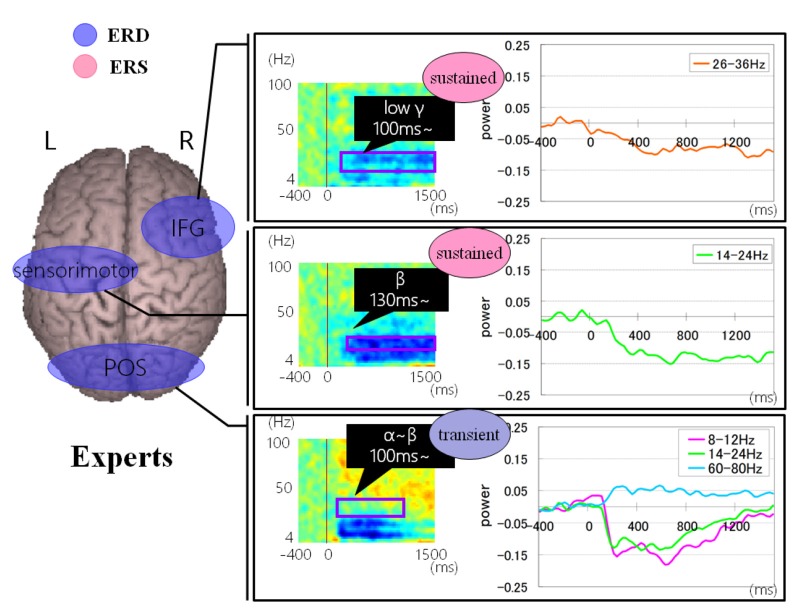
A group average of time-frequency analyses in Abacus experts during the mental multiplication experiment. The time window is between −400 and 1,500 ms; the movement onset is at 0 ms, and the frequency interval ranges from 4 Hz to 100 Hz. The red areas in the time-frequency maps indicate increases in the high gamma power (ERS), and the blue areas indicate decreases in the beta/alpha/low gamma power (ERD).

Figures 9, 10 show group average of time-frequency analyses in abacus experts and non-experts for mental multiplication experiment. For non-experts (see Figure 9), the oscillatory responses in the intraparietal sulcus are bilateral, left dominant, and frequency power is sustained. Also, the right DLPFC response was sustained. On the other hand, in experts, interestingly, the parietal response in the bilateral POS is transient. Also, both the right IFG and left sensorimotor responses were sustained (see Figure 10).

## Discussion

In the present study, we investigated calculation-related oscillatory changes using SAM group analysis and time-frequency analysis. We could elucidate the difference in calculation process between abacus experts and non-experts. In our proposed experimental paradigms, we checked if there may be a drastic difference in the strategy of calculation between abacus experts and non-experts, and if abacus experts use an imaginary abacus or abacus memory. Non-experts claim to calculate any visualized numbers using memorized multiplication or addition tables while abacus experts claim that abacus is visualized in the front of their eyes or their mind and the beads move automatically when they try to solve mathematical operations. This study aims to elucidate the difference in neural processing mechanism of mental calculation between experts and non-experts using magnetic source imaging (Della Puppa et al., 2015). Few studies tried to clarify the spatiotemporal characteristics of brain activity during addition and multiplication calculation tasks (Ishii et al., 2014; Vansteensel et al., 2014; Ueda et al., 2015).

Taking into account of temporal profiles of oscillatory changes in the first experimental paradigm, we concluded that non-experts might use serial processing; in contrast, experts may utilize parallel processing (see Supplementary Figures S11, S12). We could propose calculation processing in both abacus expert and non- experts based on our new findings and some previous studies (Hanakawa et al., 2002, 2003; Arsalidou and Taylor, 2011; Tanaka et al., 2012; Pinel and Dehaene, 2013; Amalric and Dehaene, 2017). In non-experts, from 75 ms visual processing of presented numbers start in the bilateral medial occipital, then from 150 ms figurative cognition of numbers in the inferior temporal (Dehaene et al., 1996; Pinel et al., 1999) and numeric processing in the bilateral IPS (Dehaene et al., 1996; Cohen et al., 2000; Rickard et al., 2000; Kazui et al., 2000; Bugden et al., 2019), from 200 ms inner speech in the left IFG (Dehaene et al., 1996), from 250 ms working memory in the DLPFC (Rickard et al., 2000), and finally from 400 ms addition with carrying in the IPS starts. Therefore, the calculation in non-experts is serial processing. In contrast, abacus experts calculate using parallel processing, following visual processing from 75 ms, figurative cognition in the inferior temporal, numeric processing in the IPS, and inner speech in the IFG start simultaneously from 250 ms. Table 1 shows the similarities and differences of brain activities in non-experts and abacus experts during mental operation tasks.

**Table 1:** Similarities and differences of brain activity during mental operation tasks as revealed by conjoint analysis across subjects in each group for the proposed model of calculation processing.

Regions (most significant Brodmann area) and their function	Non-experts (time-frequency characteristics)		Abacus experts (time-frequency characteristics)	
	Addition	Multiplication	Addition	Multiplication
Medial Occipital for visual processing	✓ θ ERS ~50 ms	✓ θ ERS ~50 ms	✓ θ ERS ~50 ms	✓ θ ERS ~50 ms
The medial prefrontal cortex (mPFC) for concentration (Sasaki et al., 1996; Ishii et al., 1999)	✓ θ ERS ~180 ms	✓ θ ERS ~180 ms	✓ θ ERS ~140 ms	✓ θ ERS ~140 ms
DLPFC for working memory	low γ ERD (Sustained) ~110 ms	low γ ERD (Sustained) ~110 ms	×	×
Right IPS for numerical processing	α, β, and low γ ERD (Sustained) ~110 ms	ERD (Sustained) ~110 ms	×	×
Left IPS for numerical processing	high γ ERS and α, β, and low γ ERD (Sustained) ~110 ms	high γ ERS and α, β, and low γ ERD (Sustained) ~110 ms	×	×
Sensorimotor for beads manipulation of the imaginary abacus	×	×	β ERD (Sustained) ~130 ms	β ERD (Sustained) ~130 ms
IFG for special control of moving abacus beads (Campitelli et al., 2007)	×	×	low γ ERD (Sustained) ~100 ms	low γ ERD (Sustained) ~100 ms
Bilateral POS for visual working memory to transform numbers to abacus beads (Tuladhar et al., 2007)	×	×	α, and β ERD (Transient) ~110 ms	α, and β ERD (Transient) ~110 ms

*The symbol “✓” means a common brain area for experts and non-experts. The symbol “×” means that the brain area was not activated during mental calculation*.

**Figure 11:**
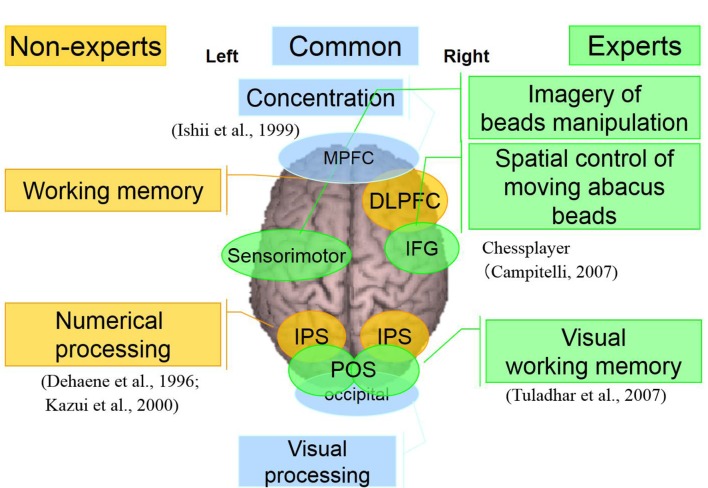
A neural processing mechanism of mental calculation based on cerebral oscillatory changes for Abacus experts and non-experts.

In both experiments where the participants were asked to perform mental operations, we observed some common brain activities in both experts and non-experts. Also, the right DLPFC and bilateral IPS were explicitly detected in non-experts. Bilateral IPS is related to numerical processing (Bugden et al., 2019), while the right DLPFC is most probably related to working memory. In experts, bilateral POS, right IFG, and left sensorimotor areas were detected specifically. POS is related to visual working memory. This response was transient, so probably used merely to transform numbers to abacus beads rather than numerical processing. Right IFG activation is reported in a Chess-players’ study (Campitelli et al., 2007). The study suggested IFG plays a crucial role in working memory of strategic spatial configuration of chess pieces. So in the present study, the right IFG may play a vital role in the strategic spatial control of moving abacus beads. The left sensorimotor area is probably related to the imagery of beads manipulation. After long time training, these areas might act as an imaginary abacus or abacus memory, so that experts do not have to logical numerical processing in left IPS, instead quickly perform complex calculation just by retrieving memorized abacus memory as if spatial pattern matching (see Supplementary Figure S25). Finally, for our proposed model of calculation processing in normal people and abacus experts, we do believe that normal people calculate logically depending on numerical processing in left IPS. In contrast, abacus experts utilize spatial processing using memorized imaginary abacus, which distributed over the bilateral hemispheres (see Figure 11).

As noted above, the present Abacus-based mental calculation experiments sought to provide a conceptual advancement. Notably, being able to find a model of calculation processing, the calculation-related area is an essential step toward understanding the brain processing mechanism, thereby leading to enhance the mathematical performance of patients with Acalculia. Although further studies are required, we would like to apply these findings (see Table 1) clinically to the less invasive neurosurgical treatments, considering not only standard calculation-related areas but also inter-individual variation including extreme brains.

Another important question still unanswered refers to the challenges of performing extremely complex calculations (5 × 5 digits). In this study, the experts have high-level mental calculation with 5–27 years’ experience, but we did not include world top-level experts. These top-level experts may show completely different brain activities, which means different neural mechanisms during the complex mental calculation. However, these experts are not likely to be able to stop mental calculation even during observation tasks because the results will automatically come to their minds instantly. It means that these top-level experts may perform mental calculations automatically independent from their will and this unanswered question should be addressed in the future.

## Conclusion

For the abacus experts in performing computations, different brain areas are involving in beads manipulation, and special control of imaginary abacus was observed. These unique findings suggested that, through an effective abacus training, the experts developed a new computational pathway by assigning number representations onto an imaginative abacus representation, through a different brain network.

We concluded that non-experts might use serial processing; in contrast, experts may utilize parallel processing. Abacus experts may acquire this processing system after long time training, and their MEG results demonstrated that calculation-related areas are simultaneously activated using abacus within the brain, and these parallel processing processes indeed significantly shorten the computation time.

## Data Availability Statement

The datasets generated and analyzed during the current study are not publicly available due to Osaka University Hospital policy but are available from the corresponding author on reasonable request.

## Ethics Statement

The studies involving human participants were reviewed and approved by The Ethics Committee at Osaka University Hospital. The experimental protocol was carried out according to the latest version of the Declaration of Helsinki. The patients/participants provided their written informed consent to participate in this study.

## Author Contributions

MH designed the study. KK and EU performed experiments and analyses. AB performed analyses, literature review, and drafted the manuscript. MH supervised the research and revised the manuscript. All authors provided critical feedback, reviewed, edited and approved the final version of the manuscript.

## Conflict of Interest

The authors declare that the research was conducted in the absence of any commercial or financial relationships that could be construed as a potential conflict of interest.

## Abbreviations

MEG: Magnetoencephalography
AMC: Abacus-based mental calculation
IPS: Intraparietal sulcus
POS: Parieto-occipital sulcus
IFG: Inferior frontal gyrus
DLPFC: Dorsolateral prefrontal cortex
mPFC: The medial prefrontal cortex
ERS: Event-related synchronization
ERD: Event-related desynchronization
SAM: Synthetic aperture magnetometry.
