# Supplementary material for: Neural Processing Mechanism of Mental Calculation Based on Cerebral Oscillatory Changes: A Comparison Between Abacus Experts and Novices
Source: Front Hum Neurosci. 2020 Apr 15;14:137. doi: 10.3389/fnhum.2020.00137 (PMC7176303; doi:10.3389/fnhum.2020.00137)
Supplement: Supplementary file 1 [file Data_Sheet_1.docx]

Supplementary Information for

**Neural Processing Mechanism of Mental Calculation Based on Cerebral Oscillatory Changes: A Comparison between Abacus Experts and Novices**

**Abdelkader Nasreddine Belkacem**^1,*^, **Kanako Kiso**^4^**, Etsuko Uokawa**^4^**, Tetsu Goto**^4^**, Shiro Yorifuji**^4^**, Masayuki Hirata**^2,3,4,5,*^

^(1)^Department of Computer and Network Engineering, College of Information Technology, United Arab Emirates University, Al Ain 15551, UAE.

^(2)^ Department of Neurological Diagnosis and Restoration, Osaka University Graduate School of Medicine, Suita, Osaka, 565-0871, Japan

^(3)^ Endowed Research Department of Clinical Neuroengineering. Global Center for Medical Engineering and Informatics, Osaka University, Osaka, 565-0871, Japan.

^(4)^Department of Neurosurgery, Osaka University Graduate School of Medicine, Suita, Osaka, 565-0871, Japan

^(5)^Center for Information and Neural Networks (CiNet), National Institute of Information and Communications Technology, and Osaka University, Suita, Osaka, 565-0871, Japan

**Contents:**

- **Spatiotemporal analyses of cerebral oscillatory changes during mental complex addition (abacus experts versus non-experts)**

Supplementary Figures 1-12

Supplementary Table 1-4

- **Spatiotemporal analyses of cerebral oscillatory changes during mental complex multiplication (abacus experts versus non-experts)**

Supplementary Figures 13-25

Supplementary Table 5-8

**Spatiotemporal analyses of cerebral oscillatory changes during mental complex addition (abacus experts versus non-experts)**

Supplementary Figures 1-12

Supplementary Table 1-4

**Supplementary Table 1.** SAM Group analysis results (Calculation tasks for non-abacus experts). The table includes frequency bands with ERS or ERD, Cluster level K-means, Pseudo-t (Non-parametric Threshold), the Family-Wise Error rate (FWE), MNI coordinates (X, Y, Z), Brodmann area, and the anatomical localization. P<0.05, corrected P value, R, right; L, left; inf., inferior; sup., superior; mid., middle.

**Supplementary Table 2.** SAM Group analysis results (Observation tasks for non-abacus experts). The table includes frequency bands with ERS or ERD, Cluster level K-means, Pseudo-t (Non-parametric Threshold), the Family-Wise Error rate (FWE), MNI coordinates (X, Y, Z), Brodmann area, and the anatomical localization. P<0.05, corrected P value, R, right; L, left; inf., inferior; sup., superior; mid., middle.

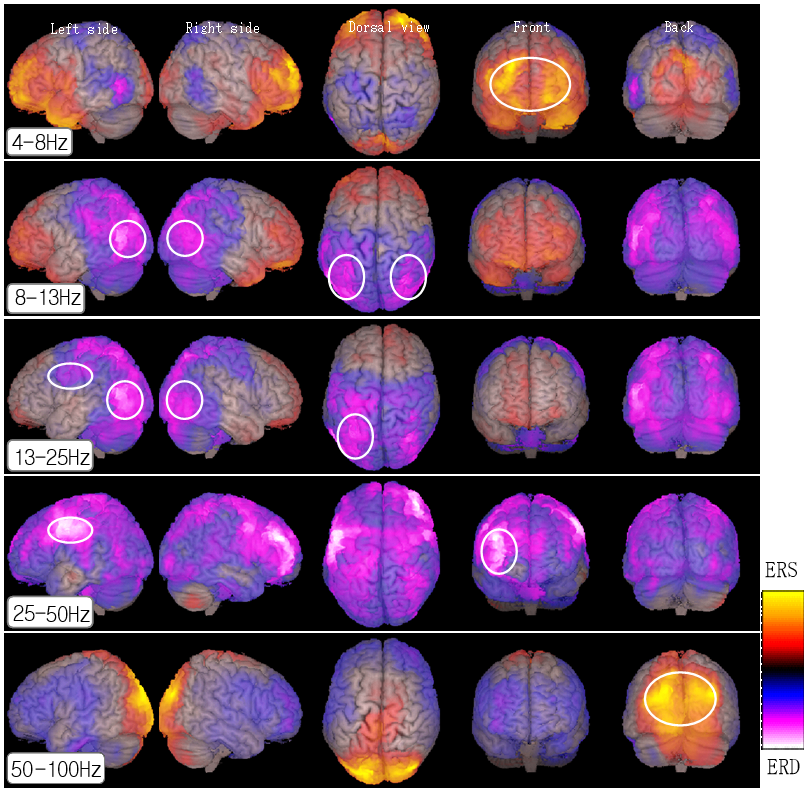


**Supplementary Figure 1.** A group average of SAM source level result of the spatial distribution of oscillatory changes for the first experiment (mental addition during calculation task) of non-abacus experts. The time window is between -1000 and 1000 ms. The frequency interval (all frequency bands) ranges from 4 Hz to 100 Hz. Magenta colour shows ERD and orange colour shows ERS. The circled areas indicate statistically-significant oscillatory changes. The significant differences observed (p<0.05, corrected) in some brain areas are surrounded by a white circle. The figure views from the left side, the right side, the upper side, the front side, and the backside are shown from the left to the right of the figure. From top to bottom, we have of θ, α, β, low γ, and the high γ bands.


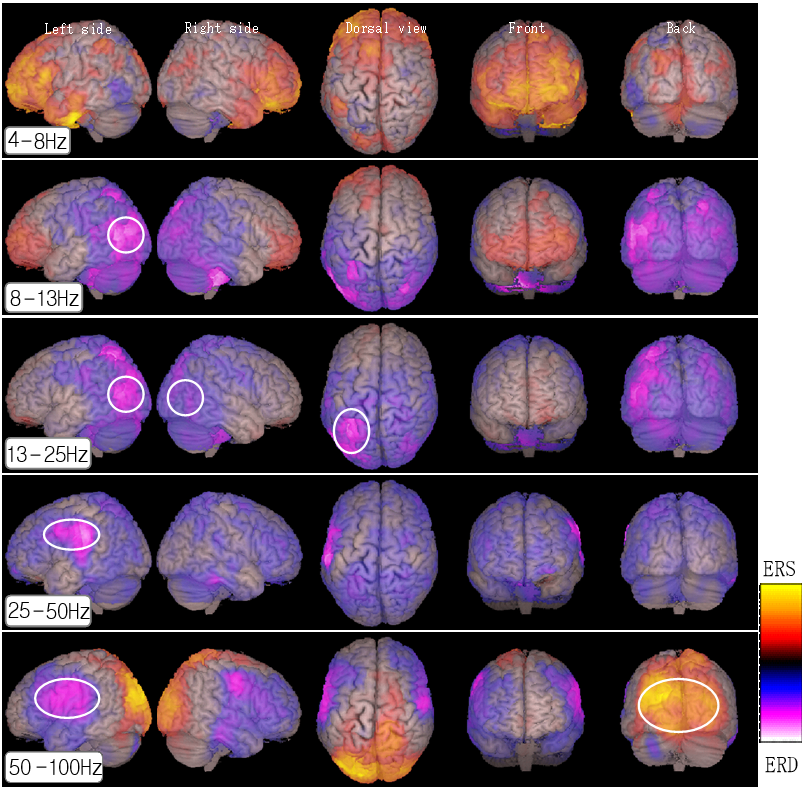


**Supplementary Figure 2.** A group average of SAM source level result of the spatial distribution of oscillatory changes for the first experiment (mental addition during observation task) of non-abacus experts. The time window is between -1000 and 1000 ms. The frequency interval (all frequency bands) ranges from 4 Hz to 100 Hz. Magenta colour shows ERD and orange colour shows ERS. The circled areas indicate statistically-significant oscillatory changes. The significant differences observed (p<0.05, corrected) in some brain areas are surrounded by a white circle. The figure views from the left side, the right side, the upper side, the front side, and the backside are shown from the left to the right of the figure. From top to bottom, we have of θ, α, β, low γ, and the high γ bands.

**Supplementary Table 3.** SAM Group analysis results (Calculation tasks for abacus experts). The table includes frequency bands with ERS or ERD, Cluster level K-means, Pseudo-t (Non-parametric Threshold), the Family-Wise Error rate (FWE), MNI coordinates (X, Y, Z), Brodmann area, and the anatomical localization. P<0.05, corrected P value, R, right; L, left; inf., inferior; sup., superior; mid., middle.

**Supplementary Table 4.**  SAM Group analysis results (Observation tasks for abacus experts). The table includes frequency bands with ERS or ERD, Cluster level K-means, Pseudo-t (Non-parametric Threshold), the Family-Wise Error rate (FWE), MNI coordinates (X, Y, Z), Brodmann area, and the anatomical localization. P<0.05, corrected P value, R, right; L, left; inf., inferior; sup., superior; mid., middle.

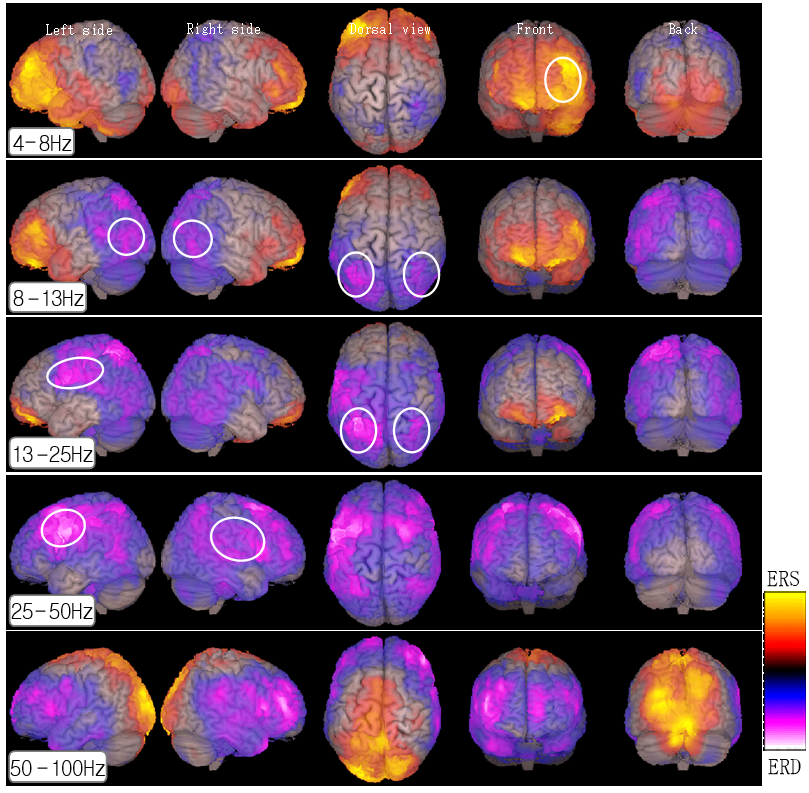


**Supplementary Figure 3.** A group average of SAM source level result of the spatial distribution of oscillatory changes for the first experiment (mental addition during calculation task) of abacus experts. The time window is between -1000 and 1000 ms. The frequency interval (all frequency bands) ranges from 4 Hz to 100 Hz. Magenta colour shows ERD and orange colour shows ERS. The circled areas indicate statistically-significant oscillatory changes. The significant differences observed (p<0.05, corrected) in some brain areas are surrounded by a white circle. The figure views from the left side, the right side, the upper side, the front side, and the backside are shown from the left to the right of the figure. From top to bottom, we have of θ, α, β, low γ, and the high γ bands.


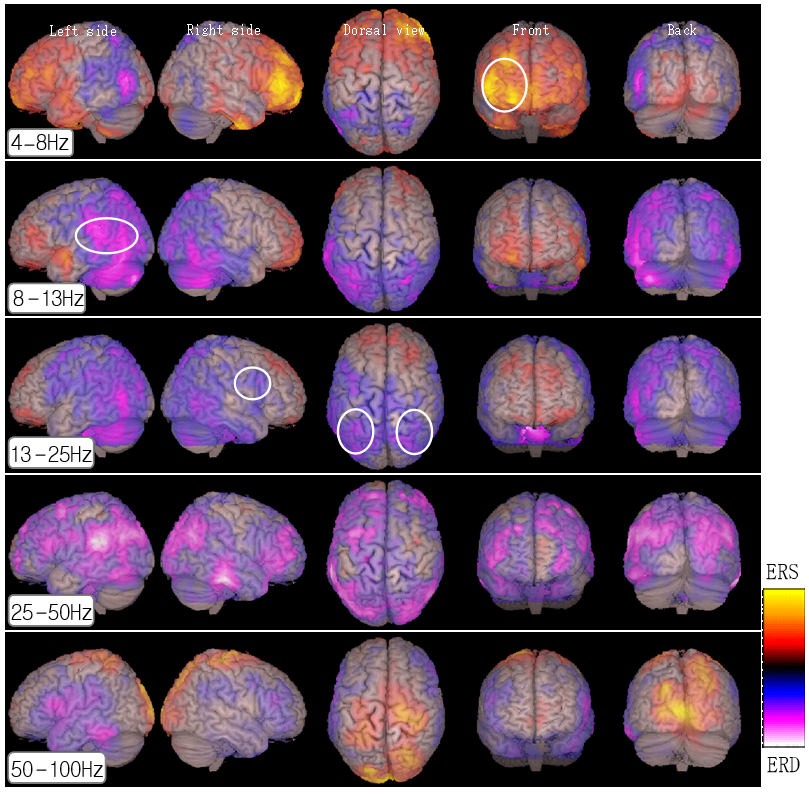


**Supplementary Figure 4.** A group average of SAM source level result of the spatial distribution of oscillatory changes for the first experiment (mental addition during observation task) of abacus experts. The time window is between -1000 and 1000 ms. The frequency interval (all frequency bands) ranges from 4 Hz to 100 Hz. Magenta colour shows ERD and orange colour shows ERS. The circled areas indicate statistically-significant oscillatory changes. The significant differences observed (p<0.05, corrected) in some brain areas are surrounded by a white circle. The figure views from the left side, the right side, the upper side, the front side, and the backside are shown from the left to the right of the figure. From top to bottom, we have of θ, α, β, low γ, and the high γ bands.


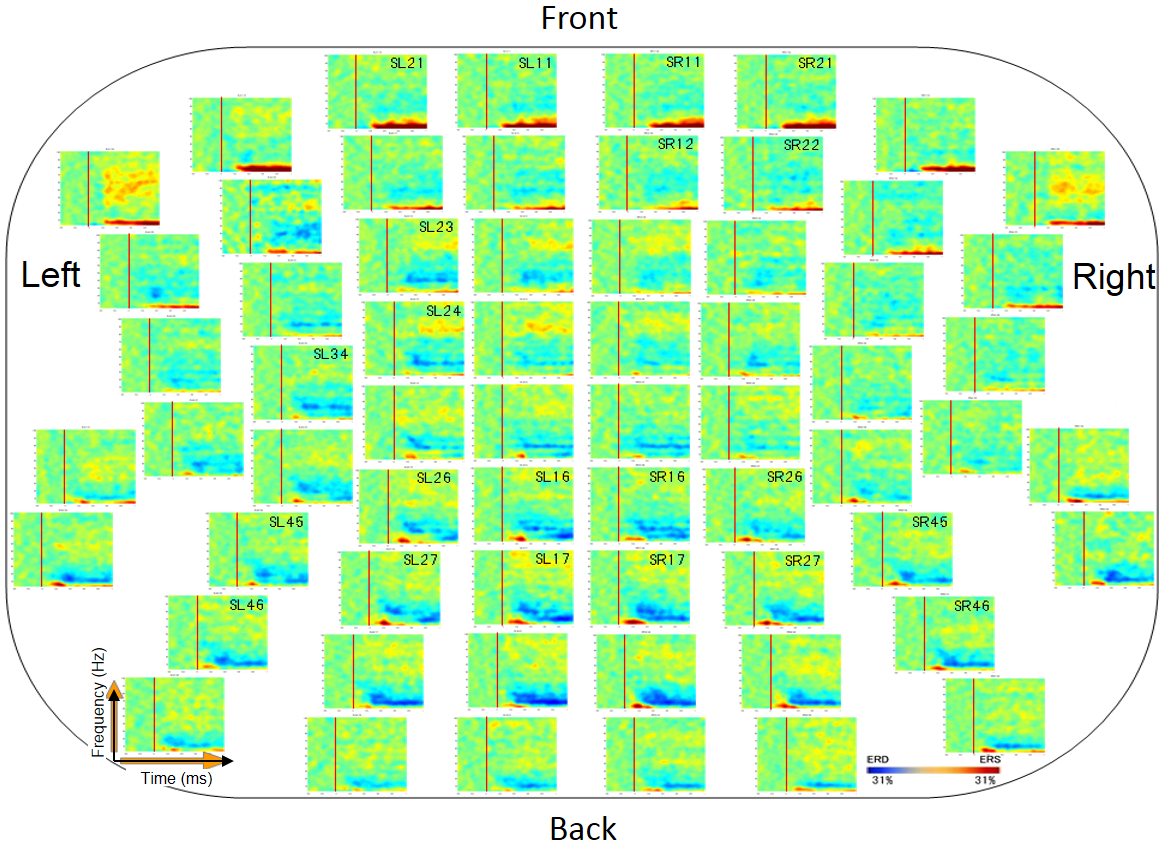


**Supplementary Figure 5.** Group average of sensor level result of time-frequency analysis of non-abacus experts during calculation task. The vertical axis represents the frequency and the horizontal axis represents the time. For color-map, blue-red shows ERD and red colour shows ERS.


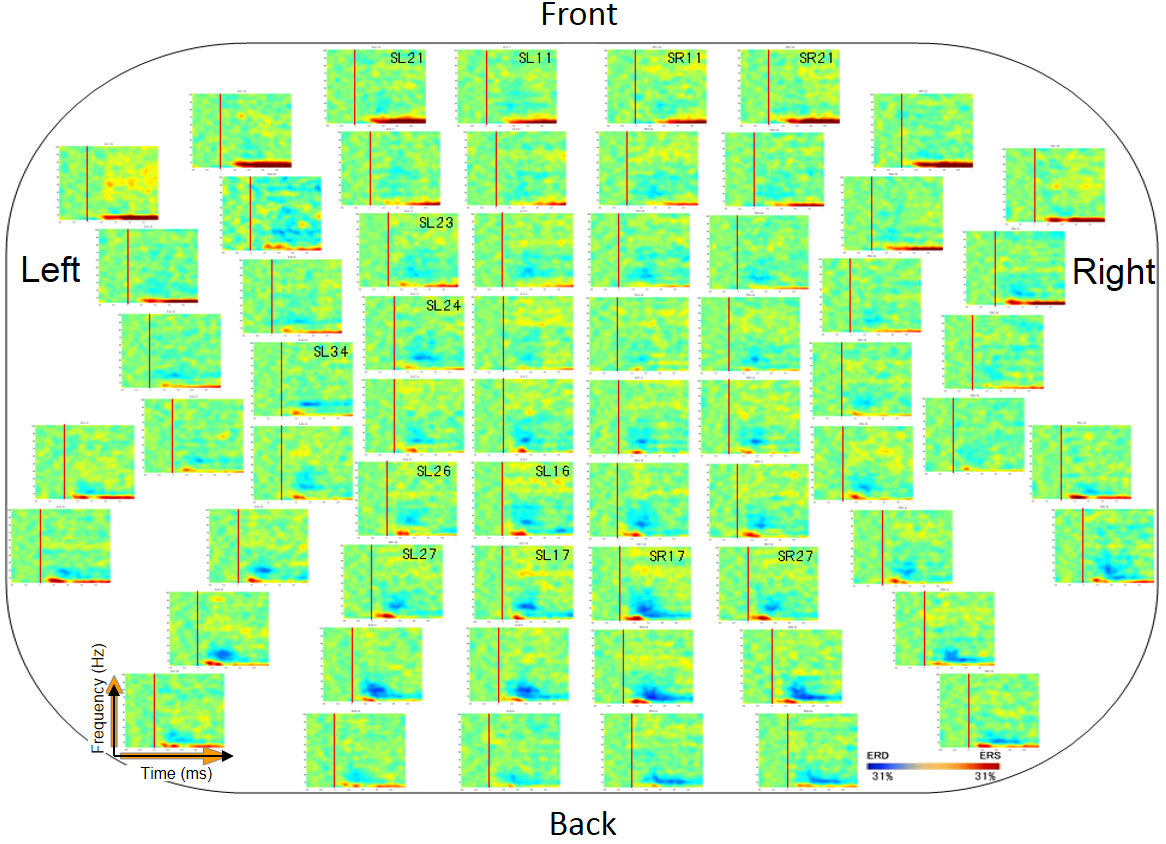


**Supplementary Figure 6.** Group average of sensor level result of time-frequency analysis of non-abacus experts during observation task. The vertical axis represents the frequency and the horizontal axis represents the time. For color-map, blue-red shows ERD and red colour shows ERS.

(A) During calculation task (b) During observation task


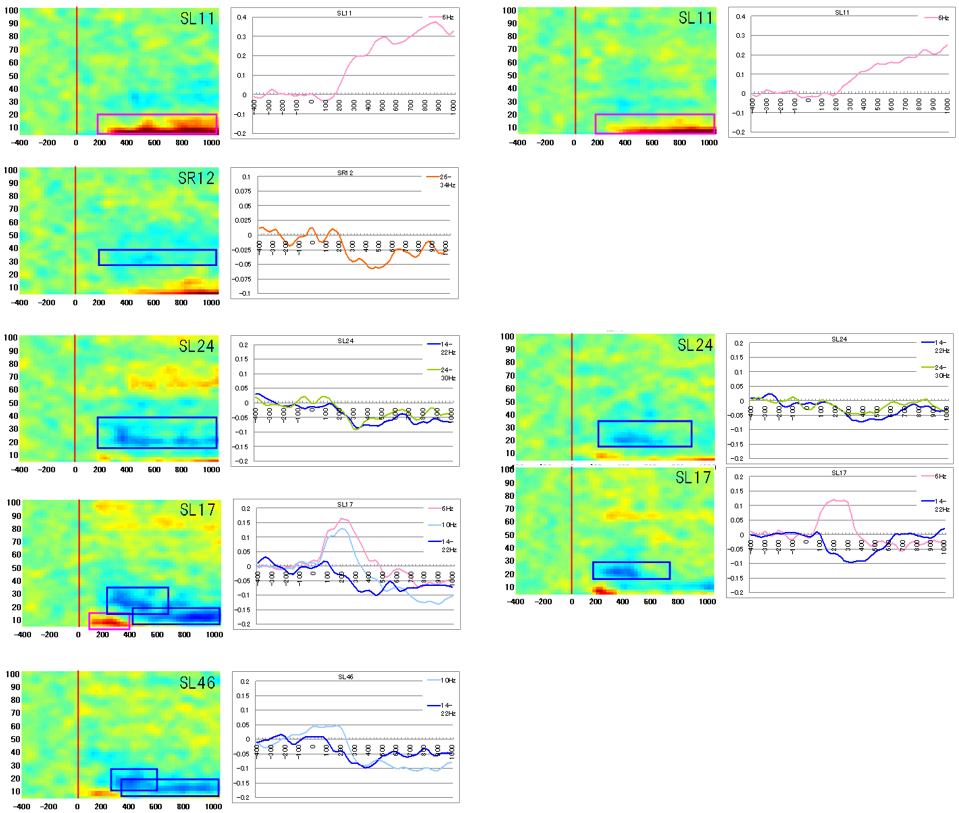


**Supplementary Figure 7.** Comparison of MEG sensors with main brain activities (ERD and ERS) for non-abacus experts during calculation and observation tasks. For color-map, blue-red shows ERD and red colour shows ERS. Fig. S7a shows MEG sensors with main brain activities (ERD and ERS) during calculation task. Fig. S7b shows MEG sensors with main brain activities (ERD and ERS) during observation task.


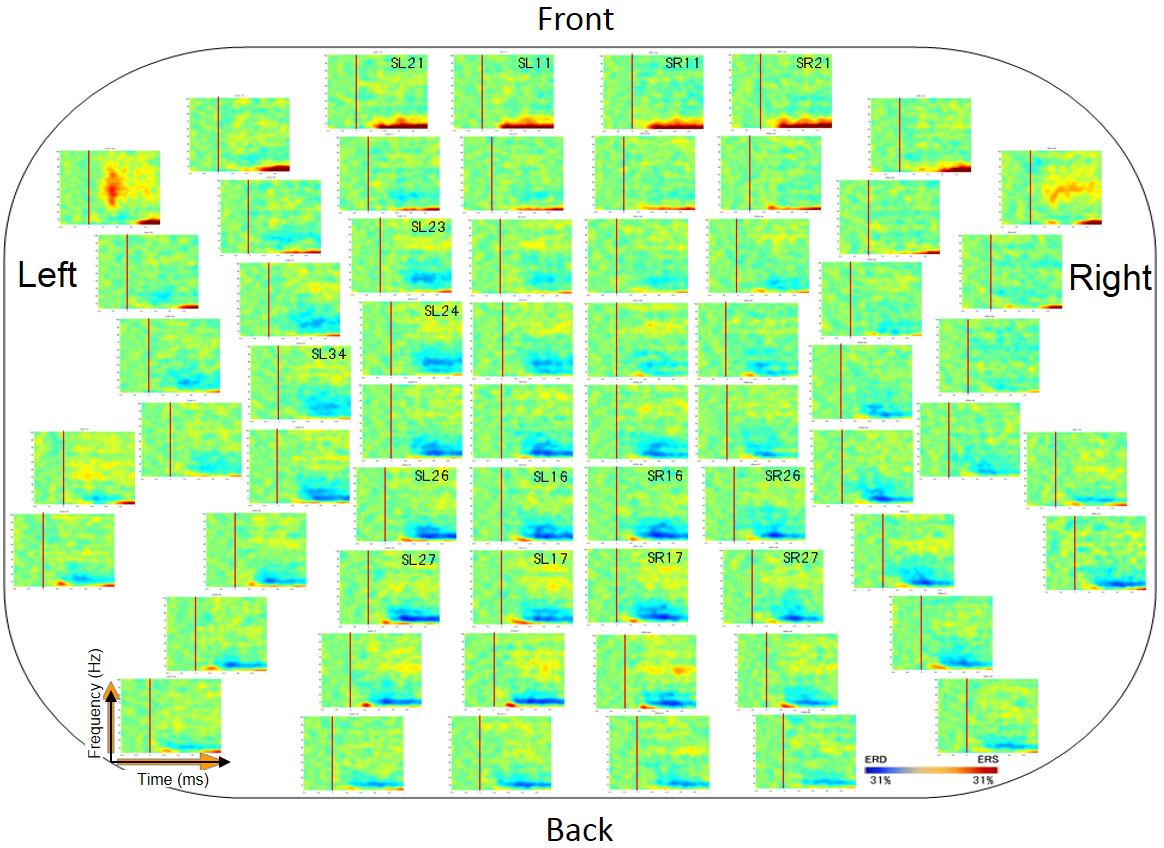


**Supplementary Figure 8.** Group average of sensor level result of time-frequency analysis of abacus experts during calculation task. The vertical axis represents the frequency and the horizontal axis represents the time. For color-map, blue-red shows ERD and red colour shows ERS.


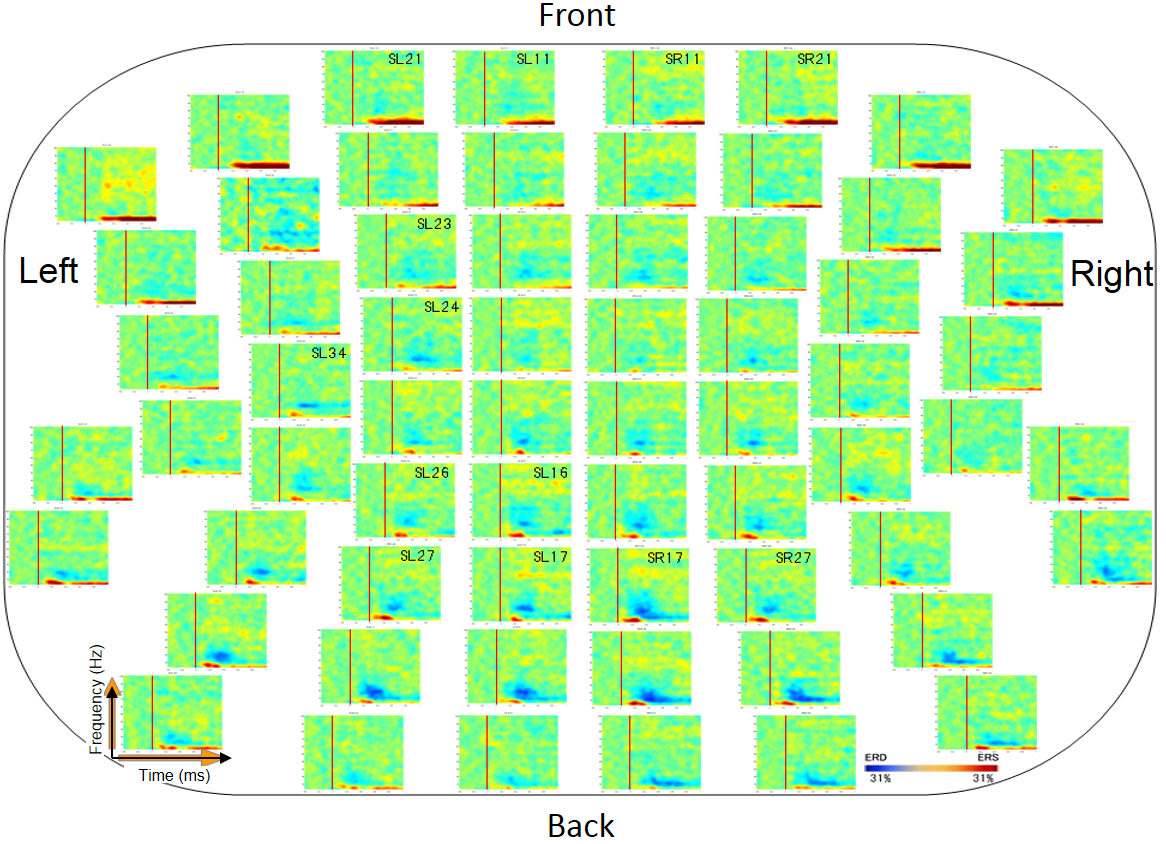


**Supplementary Figure 9.** Group average of sensor level result of time-frequency analysis of abacus experts during observation task. The vertical axis represents the frequency and the horizontal axis represents the time. For color-map, blue-red shows ERD and red colour shows ERS.

(A) During calculation task (b) During observation task


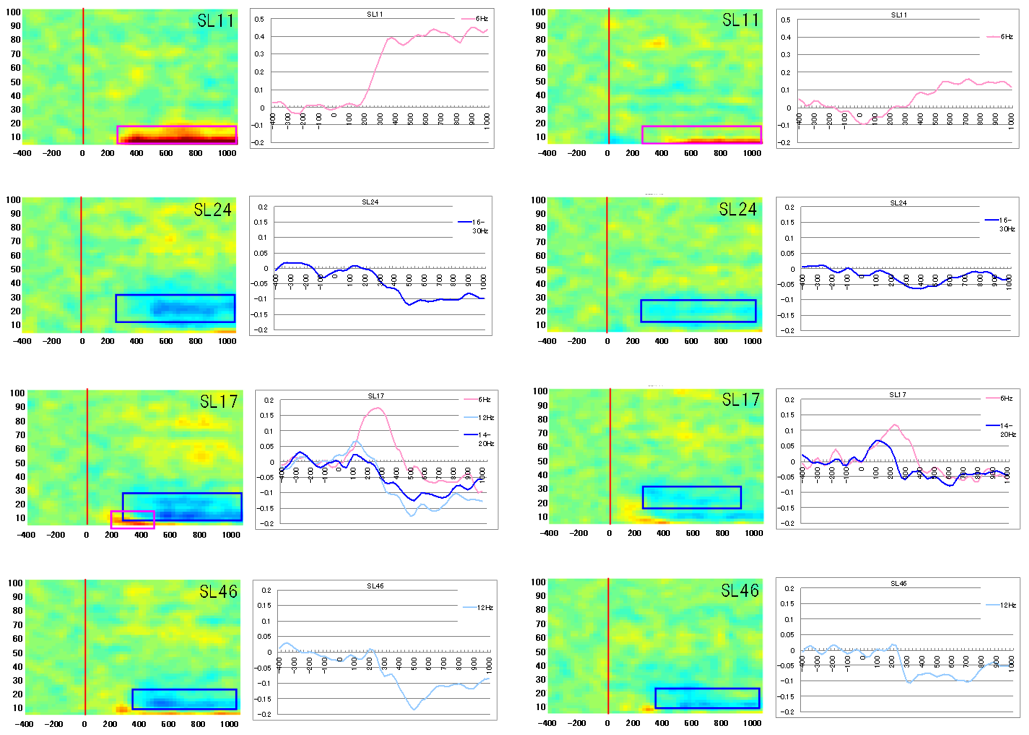


**Supplementary Figure 10.** Comparison of MEG sensors with main brain activities (ERD and ERS) for abacus experts during calculation and observation tasks. For color-map, blue-red shows ERD and red colour shows ERS. Fig. S10a shows MEG sensors with main brain activities (ERD and ERS) during calculation task. Fig. S10b shows MEG sensors with main brain activities (ERD and ERS) during observation task.


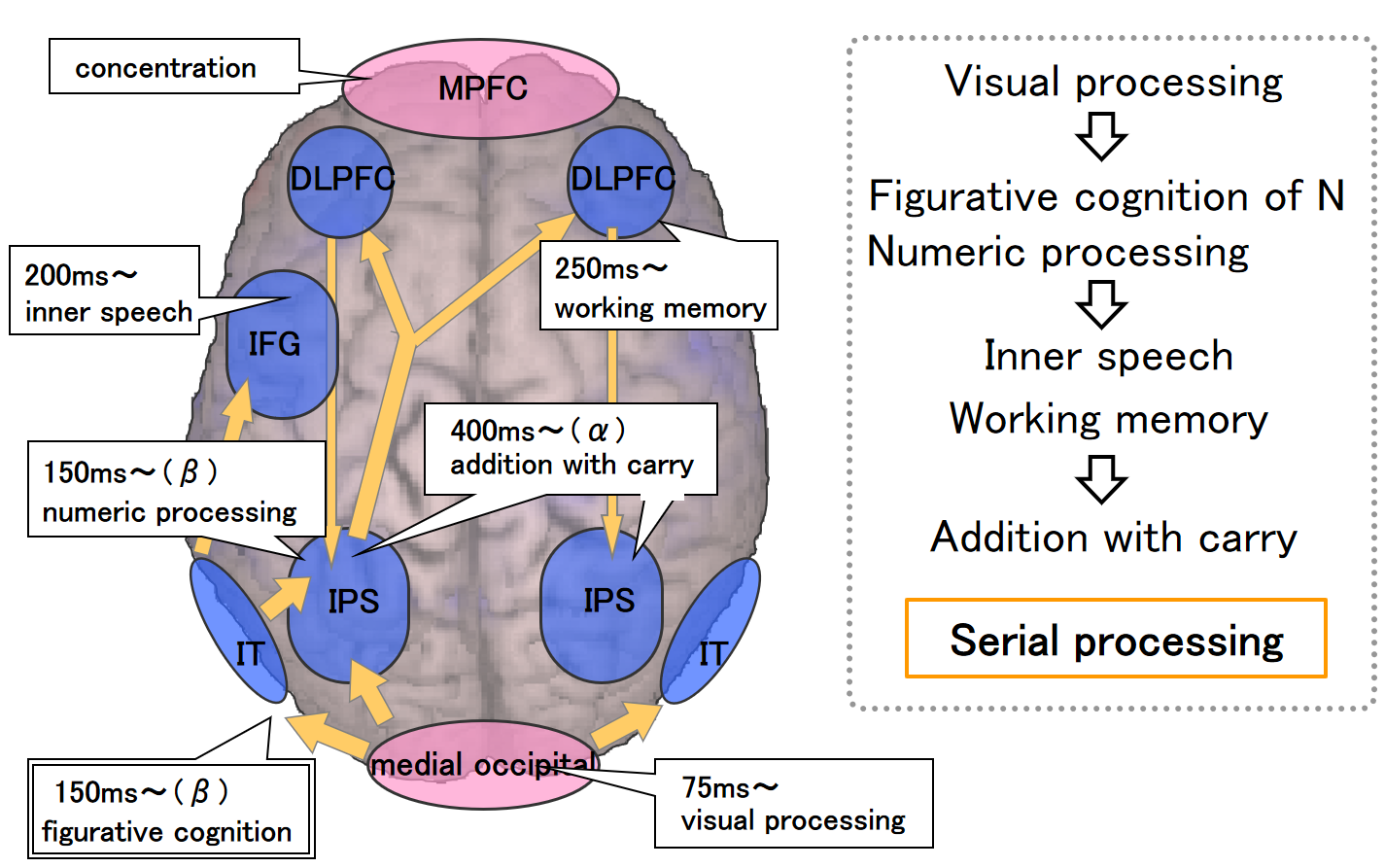


**Supplementary Figure 11.** The proposed calculation model of non-experts. Taking into account of temporal profiles of oscillatory changes, we proposed calculation processing in non- experts. From 75ms visual processing of presented numbers start in the bilateral medial occipital, then from 150ms figurative cognition of numbers in the inferior temporal and numeric processing in the bilateral IPS, from 200ms inner speech in the left IFG, from 250ms working memory in the DLPFC, and finally from 400ms addition with carry in the IPS starts. Therefore, we concluded that calculation in non-experts is serial processing. For color-map, blue-red shows ERD and pink colour shows ERS.


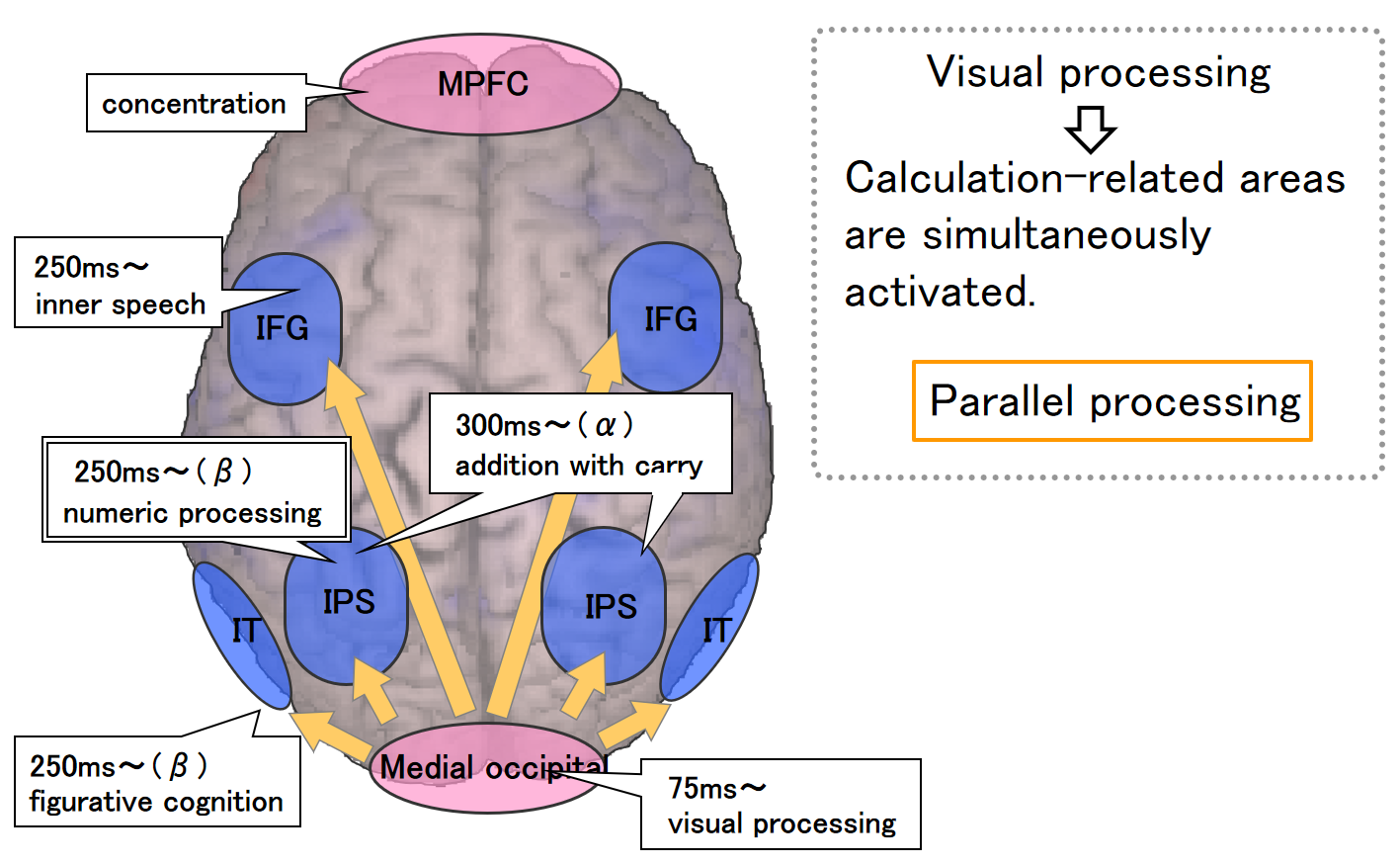


**Supplementary Figure 12.** The proposed calculation model of abacus experts. Taking into account of temporal profiles of oscillatory changes, we proposed calculation processing in experts. We found that abacus experts calculate using parallel processing. Following visual processing from 75ms, figurative cognition in the inferior temporal, numeric processing in the IPS, and inner speech in the IFG start simultaneously from 250ms. Therefore, we concluded that calculation in experts is parallel processing. For color-map, blue-red shows ERD and pink colour shows ERS.

**Spatiotemporal analyses of cerebral oscillatory changes during mental complex multiplication (abacus experts versus non-experts)**

Supplementary Figures 13-25

Supplementary Table 5-8

**Supplementary Table 5.** SAM Group analysis results (Observation tasks for non-abacus experts). The table includes frequency bands with ERS or ERD, Cluster level K-means, Pseudo-t (Non-parametric Threshold), MNI coordinates (X, Y, Z), and Brodmann area. P<0.05, corrected P value, R, right; L, left.

| Frequency | | ERS/ERD | | Cluster-level |  | Pseudo-t | MNI coordinate (mm) | | | Region (Brodmann area) | |
| --- | --- | --- | --- | --- | --- | --- | --- | --- | --- | --- | --- |
|  | | | | k |  |  | x | y | *z* |  | |
| *Ɵ* | ERS | |  | 11 652 |  | 5.81 | -12 | 28 | 42 | R | (8) |
|  |  | |  |  |  | 5.79 | -18 | 30 | 50 | R | (8) |
|  |  | |  |  |  | 5.40 | 24 | 68 | 10 | L | (10) |
|  |  | |  | 45 |  | 4.07 | -52 | 4 | -40 | R | (21) |
|  | ERD | |  | 171 |  | 4.29 | 24 | -48 | 60 | L | (7) |
| α | ERD | |  | 43559 |  | 6.23 | 28 | -54 | 48 | L | (7) |
|  |  | |  |  |  | 5.76 | 26 | -72 | 4 | L | (19) |
|  |  | |  |  |  | 5.72 | -22 | -70 | 10 | R | (17) |
|  |  | |  | 134 |  | 4.20 | -42 | -30 | 34 | R | (40) |
| β | ERD | |  | 49097 |  | 6.98 | 26 | -52 | 40 | L | (7) |
|  |  | |  |  |  | 5.47 | -20 | -64 | 30 | R | (7) |
|  |  | |  |  |  | 5.41 | 16 | -76 | 4 | L | (17) |
| low *Ƴ* | ERD | | 88880 | |  | 6.03 | 20 | -48 | 52 | L | (7) |
|  |  | |  | |  | 5.64 | -36 | 34 | 16 | R | (46) |
|  |  | |  | |  | 5.62 | -36 | 24 | 16 | R | (46) |
| high *Ƴ* | ERS | | 4518 | |  | 4.38 | 18 | -98 | 28 | R | (19) |
|  |  | |  | |  | 4.34 | -8 | -96 | 38 | R | (19) |
|  |  | |  | |  | 4.15 | 6 | -100 | 26 | L | (19) |
|  |  | | 989 | |  | 4.26 | 22 | -62 | 66 | L | (7) |

**Supplementary Table 6.** SAM Group analysis results (Observation tasks for abacus experts). The table includes frequency bands with ERS or ERD, Cluster level K-means, Pseudo-t (Non-parametric Threshold), MNI coordinates (X, Y, Z), and Brodmann area. P<0.05, corrected P value, R, right; L, left.

| Frequency | | ERS/ERD | | Cluster-level |  | Pseudo-t | MNI coordinate (mm) | | | Region (Brodmann area) | |
| --- | --- | --- | --- | --- | --- | --- | --- | --- | --- | --- | --- |
|  | | | | k |  |  | x | y | *z* |  | |
| *Ɵ* | ERS | |  | 963 |  | 4.97 | 30 | 54 | 38 | L | (9) |
|  |  | |  |  |  | 4.67 | 42 | 50 | 30 | L | (9) |
|  |  | |  |  |  | 4.63 | 52 | 40 | 12 | L | (46) |
|  |  | |  | 97 |  | 4.25 | 4 | 40 | 38 | L | (9) |
|  |  | |  |  |  | 4.09 | 10 | 40 | 30 | L | (9) |
| α | ERD | |  | 778 |  | 5.53 | 30 | -56 | 52 | L | (7) |
|  |  | |  |  |  | 5.23 | 26 | -60 | 60 | L | (7) |
|  |  | |  | 69 |  | 5.42 | -32 | -62 | 66 | R | (7) |
|  |  | |  |  |  | 4.48 | -22 | -60 | 56 | R | (7) |
|  |  | |  | 144 |  | 4.84 | 22 | -88 | -22 | L | (18) |
|  |  | |  |  |  | 4.51 | 18 | -78 | -16 | L | (18) |
|  |  | |  | 234 |  | 4.82 | -28 | -64 | 38 | R | (7) |
|  |  | |  |  |  | 4.71 | -24 | -66 | 30 | R | (7) |
|  |  | |  | 36 |  | 4.54 | 34 | -50 | -8 | L | (19) |
| β | ERD | |  | 8477 |  | 6.55 | 22 | -52 | 50 | L | (7) |
|  |  | |  |  |  | 5.77 | -22 | -50 | 54 | R | (7) |
|  |  | |  |  |  | 4.94 | -22 | -68 | 30 | R | (7) |
|  |  | |  | 24 |  | 4.40 | -44 | -86 | 2 | R | (19) |
|  |  | |  |  |  | 4.23 | -38 | -80 | 0 | R | (19) |
|  |  | |  | 33 |  | 4.31 | -32 | -54 | -6 | R | (37) |
|  |  | |  |  |  | 4.28 | -28 | -50 | -16 | R | (37) |
| low *Ƴ* | ERD | | 5785 | |  | 4.82 | -24 | -50 | 68 | R | (7) |
|  |  | |  | |  | 4.79 | 30 | -44 | 48 | L | (7) |
|  |  | |  | |  | 4.72 | 20 | -48 | 54 | L | (7) |
|  |  | | 71 | |  | 4.10 | 32 | 16 | 28 | L | (46) |
|  |  | | 239 | |  | 4.06 | -16 | 36 | 6 | R | (24) |
| high *Ƴ* | ERS | | 985 | |  | 4.64 | -24 | -96 | 34 | R | (19) |
|  |  | |  | |  | 4.27 | -32 | -94 | 22 | R | (19) |

**Supplementary Table 7.** SAM Group analysis results (Calculation tasks for non-abacus experts). The table includes frequency bands with ERS or ERD, Cluster level K-means, Pseudo-t (Non-parametric Threshold), MNI coordinates (X, Y, Z), and Brodmann area. P<0.05, corrected P value, R, right; L, left.

| Frequency | | ERS/ERD | | Cluster-level |  | Pseudo-t | MNI coordinate (mm) | | | Region (Brodmann area) | |
| --- | --- | --- | --- | --- | --- | --- | --- | --- | --- | --- | --- |
|  | | | | k |  |  | x | y | *z* |  | |
| *Ɵ* | ERS | |  | 2562 |  | 6.15 | 24 | 56 | 38 | L | (9) |
|  |  | |  |  |  | 5.67 | 34 | 50 | 12 | L | (10 |
|  |  | |  |  |  | 5.23 | 26 | 36 | 26 | L | (10) |
|  |  | |  | 1632 |  | 5.88 | -16 | 56 | -6 | R | (10) |
|  |  | |  |  |  | 4.99 | -20 | 68 | -6 | R | (10) |
|  |  | |  | 103 |  | 5.24 | -58 | 34 | 2 | R | (45) |
|  |  | |  | 164 |  | 5.17 | -10 | 66 | 26 | R | (10) |
|  |  | |  | 115 |  | 5.04 | -38 | 58 | 20 | R | (10) |
|  |  | |  |  |  | 4.68 | -40 | 52 | 26 | R | (10) |
|  |  | |  | 92 |  | 4.77 | -36 | 6 | -38 | R | (21) |
|  |  | |  | 21 |  | 4.63 | -36 | 42 | 38 | R | (9) |
|  |  | |  | 43 |  | 4.59 | -8 | 6 | 14 | R | (25) |
| α | ERS | |  | 174 |  | 4.75 | 18 | 56 | 34 | L | (9) |
|  | ERD | |  | 13577 |  | 6.25 | -28 | -74 | 20 | R | (19) |
|  |  | |  |  |  | 6.21 | -10 | -78 | 14 | R | (18) |
|  |  | |  |  |  | 6.18 | -20 | -72 | 18 | R | (31) |
| β | ERD | |  | 31479 |  | 5.94 | 16 | -66 | 26 | L | (31) |
|  |  | |  |  |  | 5.89 | 48 | -16 | 22 | L | (1,2,3) |
|  |  | |  |  |  | 5.86 | 48 | -10 | 30 | L | (6) |
|  |  | |  | 97 |  | 4.17 | -40 | -92 | 18 | R | (19) |
| low *Ƴ* | ERD | |  | 60093 |  | 8.57 | -46 | 2 | 20 | R | (44) |
|  |  | |  |  |  | 6.11 | 10 | -46 | 24 | L | (23) |
| high *Ƴ* | ERS | | 8603 | |  | 6.09 | 16 | -98 | 28 | L | (19) |
|  |  | |  | |  | 5.82 | 2 | -98 | 26 | L | (19) |
|  |  | |  | |  | 5.04 | 12 | -86 | 2 | L | (17) |
|  | ERD | | 293 | |  | 4.01 | -60 | 16 | 38 | R | (44) |
|  |  | |  | |  | 3.96 | -48 | 4 | 34 | R | (44) |

**Supplementary Table 8.** SAM Group analysis results (Calculation tasks for abacus experts). The table includes frequency bands with ERS or ERD, Cluster level K-means, Pseudo-t (Non-parametric Threshold), MNI coordinates (X, Y, Z), and Brodmann area. P<0.05, corrected P value, R, right; L, left.

| Frequency | | ERS/ERD | | Cluster-level |  | Pseudo-t | MNI coordinate (mm) | | | Region (Brodmann area) | |
| --- | --- | --- | --- | --- | --- | --- | --- | --- | --- | --- | --- |
|  | | | | k |  |  | x | y | *z* |  | |
| *Ɵ* | ERS | |  | 853 |  | 5.29 | -12 | 42 | 12 | R | (32) |
|  |  | |  |  |  | 5.27 | -24 | 32 | 2 | R | (45) |
|  |  | |  |  |  | 5.27 | -30 | 28 | 8 | R | (45) |
|  |  | |  | 38 |  | 4.86 | -4 | 58 | 40 | R | (9) |
| α | ERD | |  | 13782 |  | 6.06 | 38 | -56 | 26 | L | (39) |
|  |  | |  |  |  | 5.74 | 20 | -54 | 50 | L | (7) |
|  |  | |  |  |  | 5.71 | 16 | -62 | 50 | L | (7) |
|  |  | |  | 119 |  | 4.56 | -8 | -100 | 26 | R | (19) |
|  |  | |  |  |  | 4.25 | -8 | -90 | 20 | R | (18) |
|  |  | |  | 40 |  | 4.55 | -36 | -96 | -2 | R | (18) |
| β | ERD | |  | 19108 |  | 6.57 | 32 | -56 | 44 | L | (7) |
|  |  | |  |  |  | 6.21 | 20 | -66 | 34 | L | (7) |
|  |  | |  |  |  | 6.18 | 34 | -56 | 30 | L | (39) |
|  |  | |  | 56 |  | 4.04 | 48 | 2 | 28 | L | (44) |
|  |  | |  |  |  | 3.99 | 48 | -6 | 26 | L | (6) |
|  |  | |  | 21 |  | 3.95 | -20 | -46 | 62 | R | (5) |
| low *Ƴ* | ERD | |  | 5871 |  | 5.79 | 24 | -58 | 46 | L | (7) |
|  |  | |  |  |  | 5.68 | 22 | -64 | 54 | L | (7) |
|  |  | |  |  |  | 4.92 | 38 | -52 | 24 | L | (39) |
|  |  | |  | 1465 |  | 4.83 | -26 | -60 | 44 | R | (7) |
|  |  | |  |  |  | 4.13 | -46 | -72 | 38 | R | (40) |
|  |  | |  | 252 |  | 4.54 | 48 | -6 | 28 | L | (4) |
|  |  | |  | 94 |  | 4.32 | -30 | -4 | 38 | R | (9) |
| high *Ƴ* | ERS | | 12569 | |  | 5.49 | 16 | -80 | 12 | L | (17) |
|  |  | |  | |  | 5.22 | 8 | -82 | -4 | L | (18) |
|  |  | |  | |  | 5.20 | 12 | -82 | -12 | L | (18) |

**
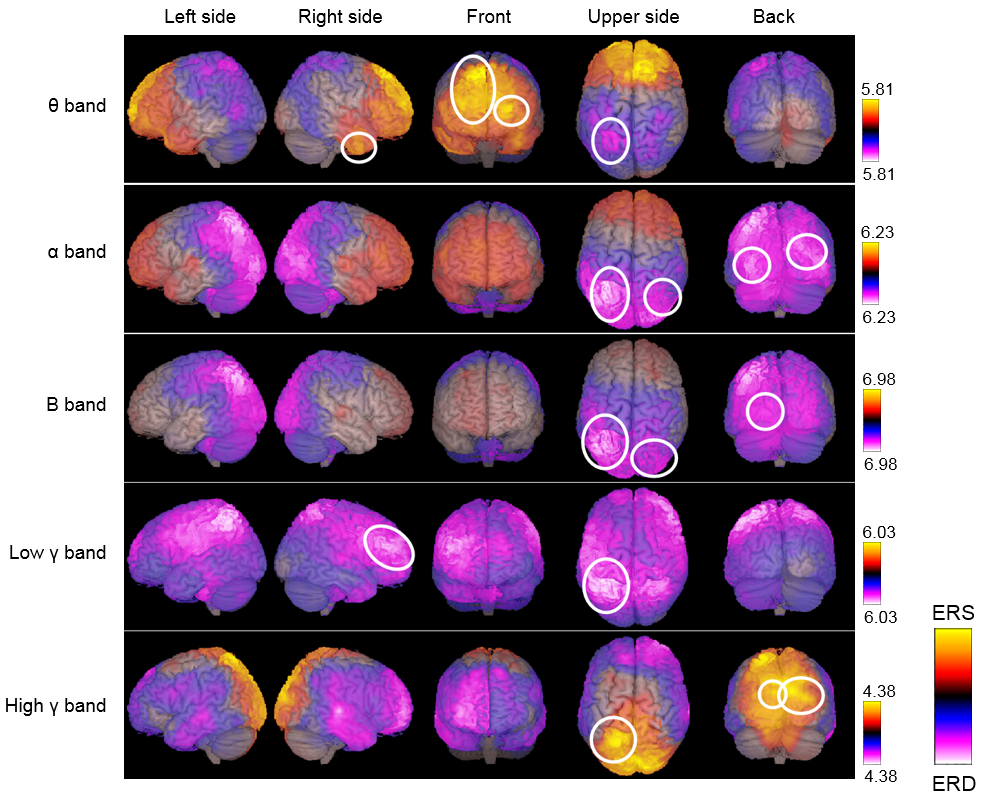
**

**Supplementary Figure 13.** A group average of SAM source level result of the spatial distribution of oscillatory changes for the first experiment (mental multiplication during calculation task) of non-abacus experts. The time window is between -1000 and 1000 ms. The frequency interval (all frequency bands) ranges from 4 Hz to 100 Hz. Magenta colour shows ERD and orange colour shows ERS. The circled areas indicate statistically-significant oscillatory changes. The significant differences observed (p<0.05, corrected) in some brain areas are surrounded by a white circle. The figure views from the left side, the right side, the upper side, the front side, and the backside are shown from the left to the right of the figure. From top to bottom, we have of θ, α, β, low γ, and the high γ bands.

**
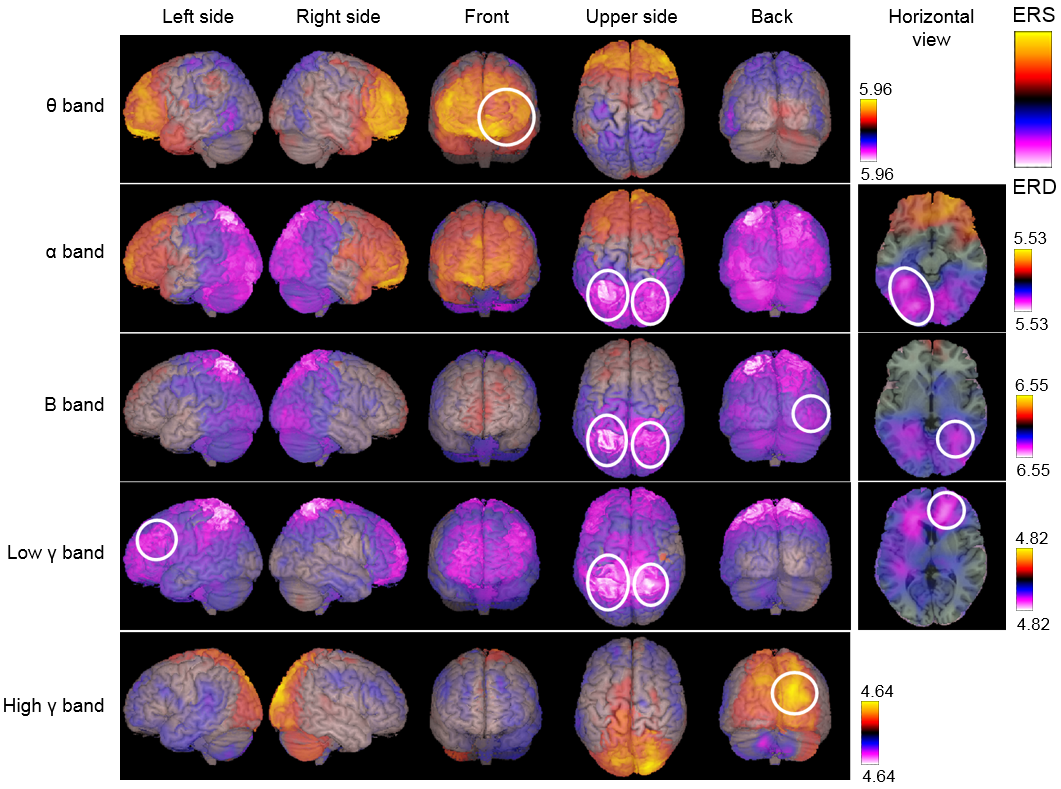
**

**Supplementary Figure 14.** A group average of SAM source level result of the spatial distribution of oscillatory changes for the first experiment (mental multiplication during observation task) of non-abacus experts. The time window is between -1000 and 1000 ms. The frequency interval (all frequency bands) ranges from 4 Hz to 100 Hz. Magenta colour shows ERD and orange colour shows ERS. The circled areas indicate statistically-significant oscillatory changes. The significant differences observed (p<0.05, corrected) in some brain areas are surrounded by a white circle. The figure views from the left side, the right side, the upper side, the front side, and the backside are shown from the left to the right of the figure. From top to bottom, we have of θ, α, β, low γ, and the high γ bands.

**
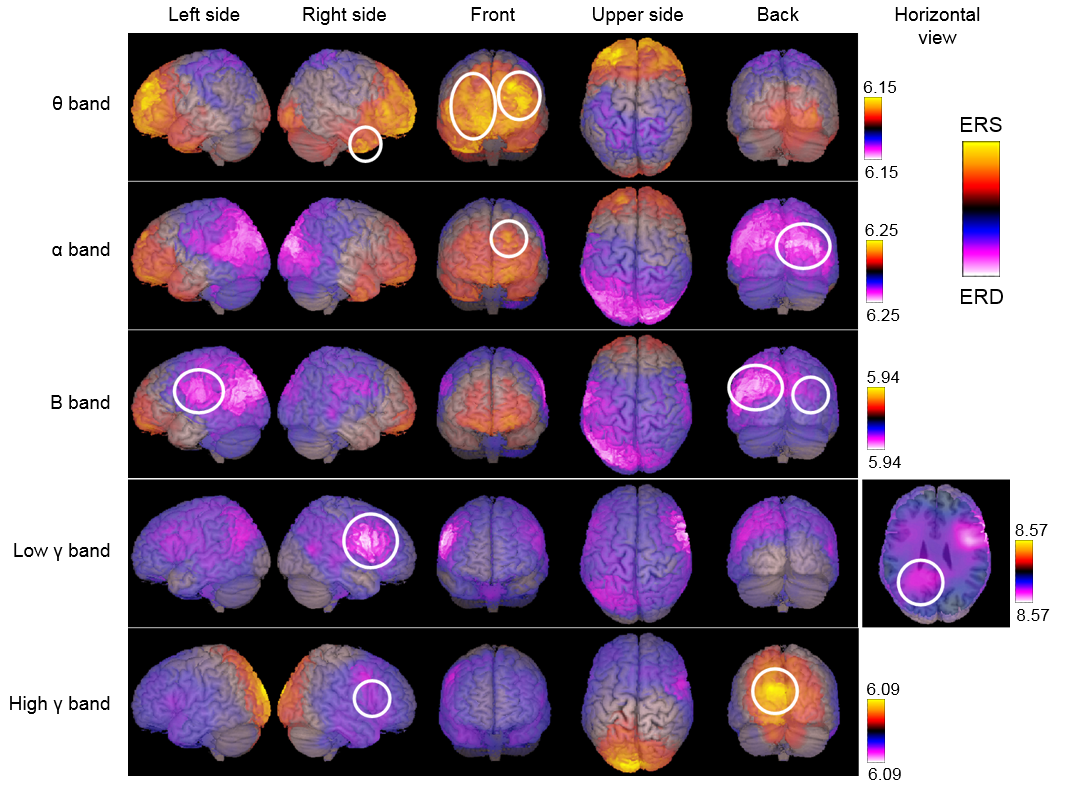
**

**Supplementary Figure 15.** A group average of SAM source level result of the spatial distribution of oscillatory changes for the first experiment (mental multiplication during calculation task) of abacus experts. The time window is between -1000 and 1000 ms. The frequency interval (all frequency bands) ranges from 4 Hz to 100 Hz. Magenta colour shows ERD and orange colour shows ERS. The circled areas indicate statistically-significant oscillatory changes. The significant differences observed (p<0.05, corrected) in some brain areas are surrounded by a white circle. The figure views from the left side, the right side, the upper side, the front side, and the backside are shown from the left to the right of the figure. From top to bottom, we have of θ, α, β, low γ, and the high γ bands.

**
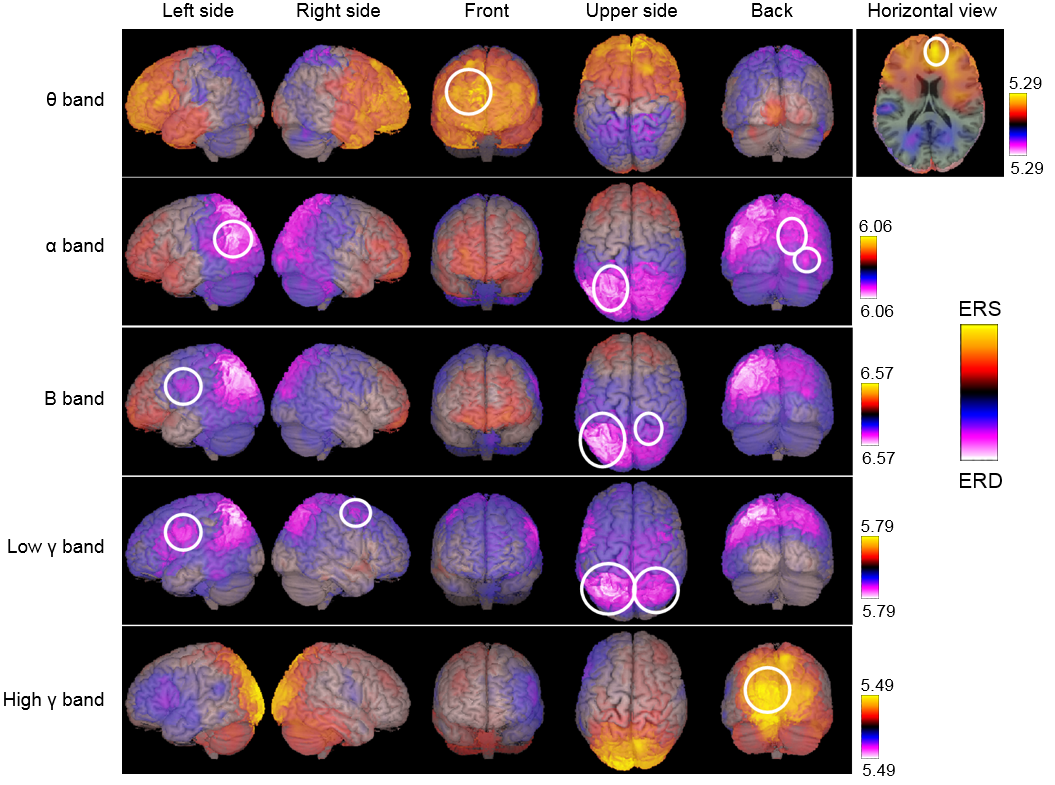
**

**Supplementary Figure 16.** A group average of SAM source level result of the spatial distribution of oscillatory changes for the first experiment (mental multiplication during observation task) of abacus experts. The time window is between -1000 and 1000 ms. The frequency interval (all frequency bands) ranges from 4 Hz to 100 Hz. Magenta colour shows ERD and orange colour shows ERS. The circled areas indicate statistically-significant oscillatory changes. The significant differences observed (p<0.05, corrected) in some brain areas are surrounded by a white circle. The figure views from the left side, the right side, the upper side, the front side, and the backside are shown from the left to the right of the figure. From top to bottom, we have of θ, α, β, low γ, and the high γ bands.

**
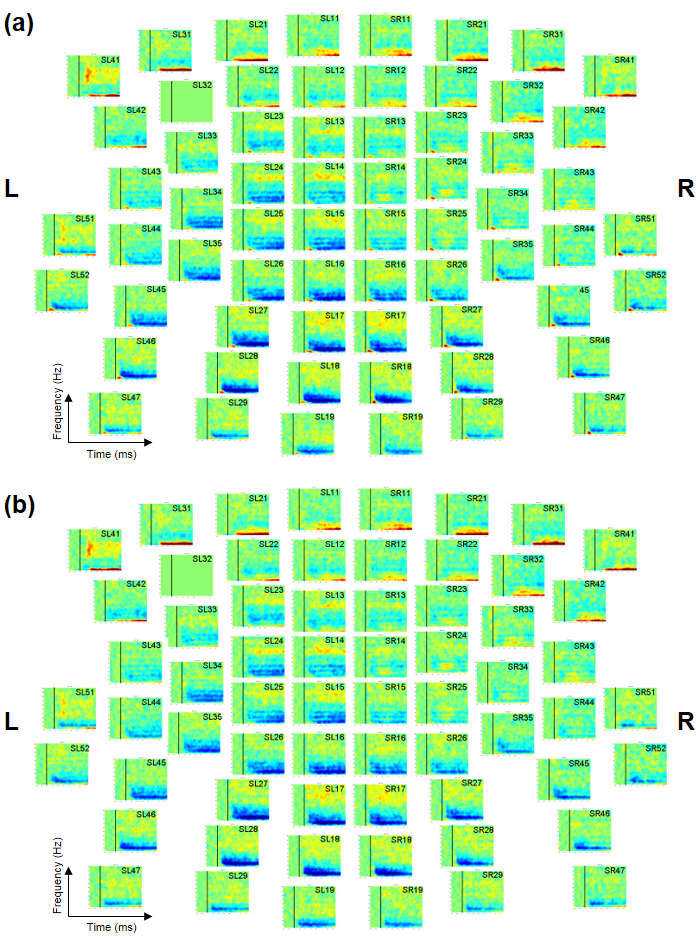
**

**Supplementary Figure 17.** Group average of sensor level result of time-frequency analysis of non-abacus experts during calculation task. The vertical axis represents the frequency and the horizontal axis represents the time. For color-map, blue-red shows ERD and red colour shows ERS. (a) Spectrum of all sensors displaying both evoked responses and induced responses. (b) Spectrograms of all sensors showing only induced responses.

**
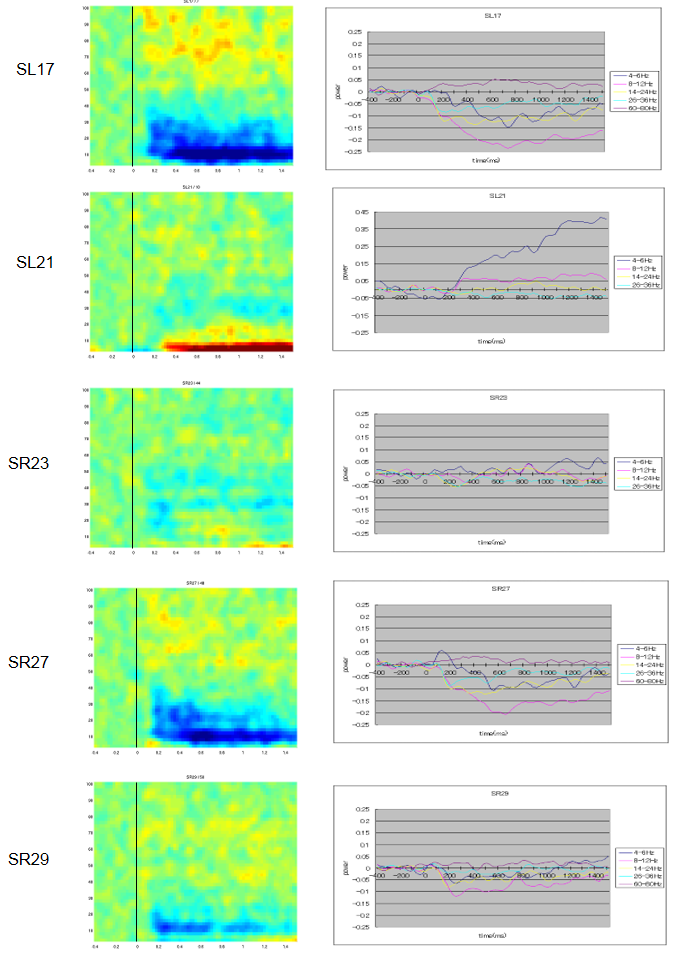
**

**Supplementary Figure 18.** MEG sensors with main brain activities (ERD and ERS) for non-abacus experts during calculation task. For color-map, blue-red shows ERD and red colour shows ERS.

**
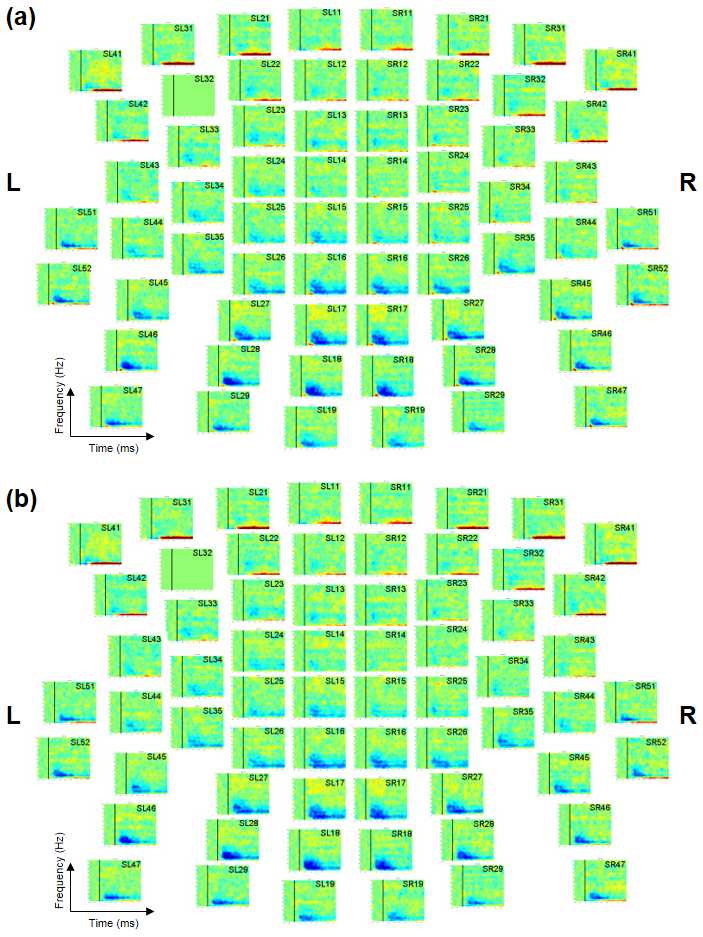
**

**Supplementary Figure 19.** Group average of sensor level result of time-frequency analysis of non-abacus experts during observation task. The vertical axis represents the frequency and the horizontal axis represents the time. For color-map, blue-red shows ERD and red colour shows ERS. (a) Spectrum of all sensors displaying both evoked responses and induced responses. (b) Spectrograms of all sensors showing only induced responses.

**
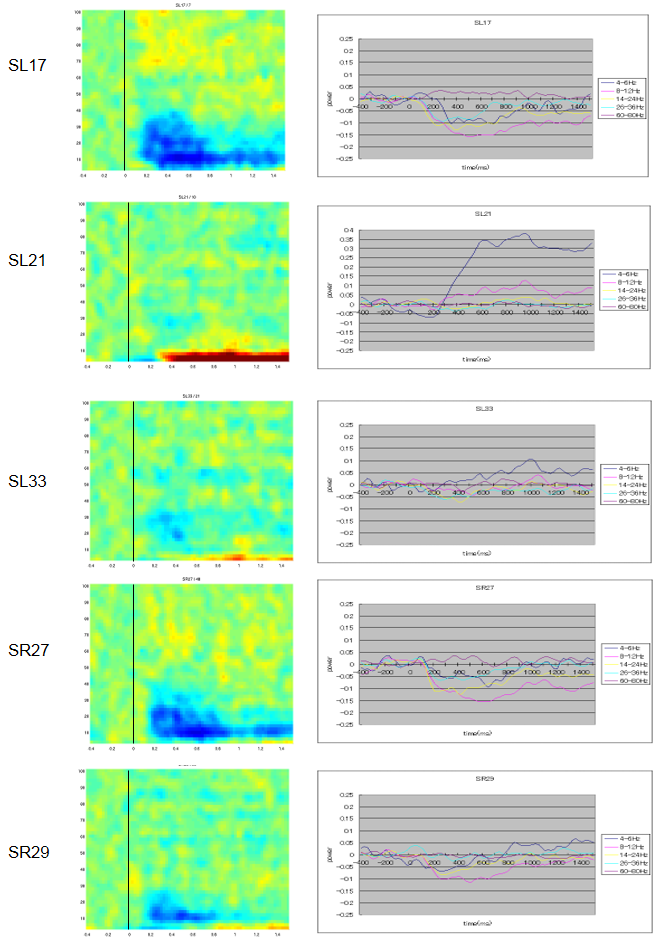
**

**Supplementary Figure 20.** MEG sensors with main brain activities (ERD and ERS) for non-abacus experts during observation task. For color-map, blue-red shows ERD and red colour shows ERS.

**
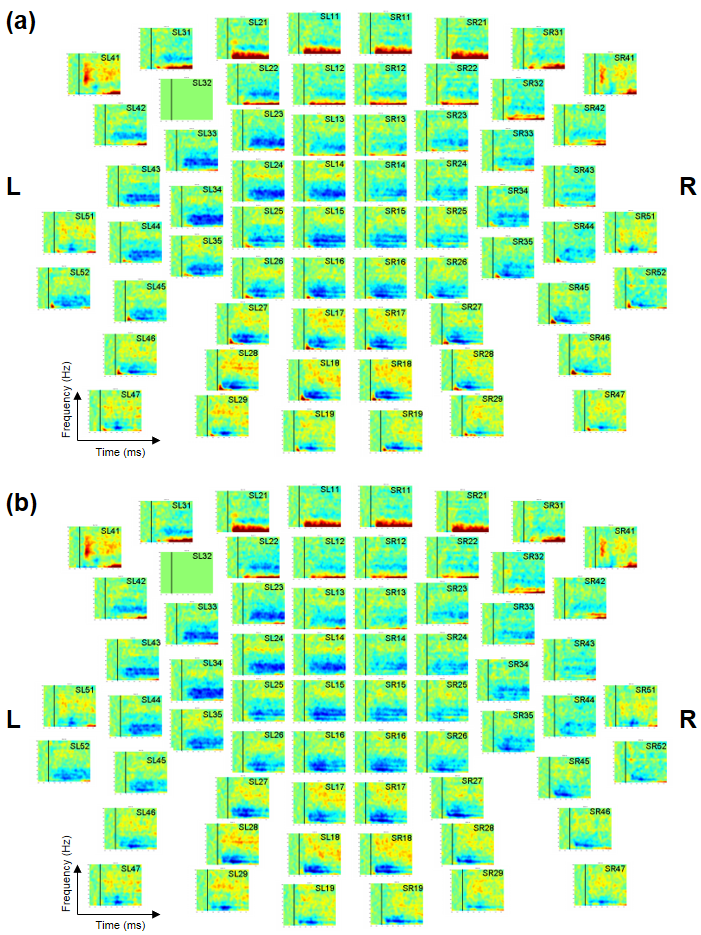
**

**Supplementary Figure 21.** Group average of sensor level result of time-frequency analysis of abacus experts during calculation task. The vertical axis represents the frequency and the horizontal axis represents the time. For color-map, blue-red shows ERD and red colour shows ERS. (a) Spectrum of all sensors displaying both evoked responses and induced responses. (b) Spectrograms of all sensors showing only induced responses.

**
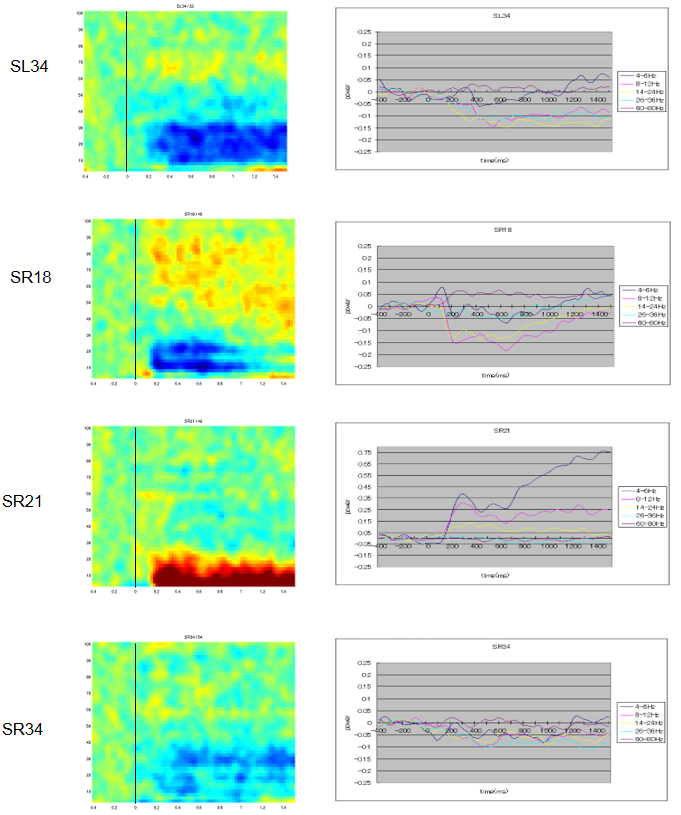
**

**Supplementary Figure 22.** MEG sensors with main brain activities (ERD and ERS) for abacus experts during calculation task. For color-map, blue-red shows ERD and red colour shows ERS.

**
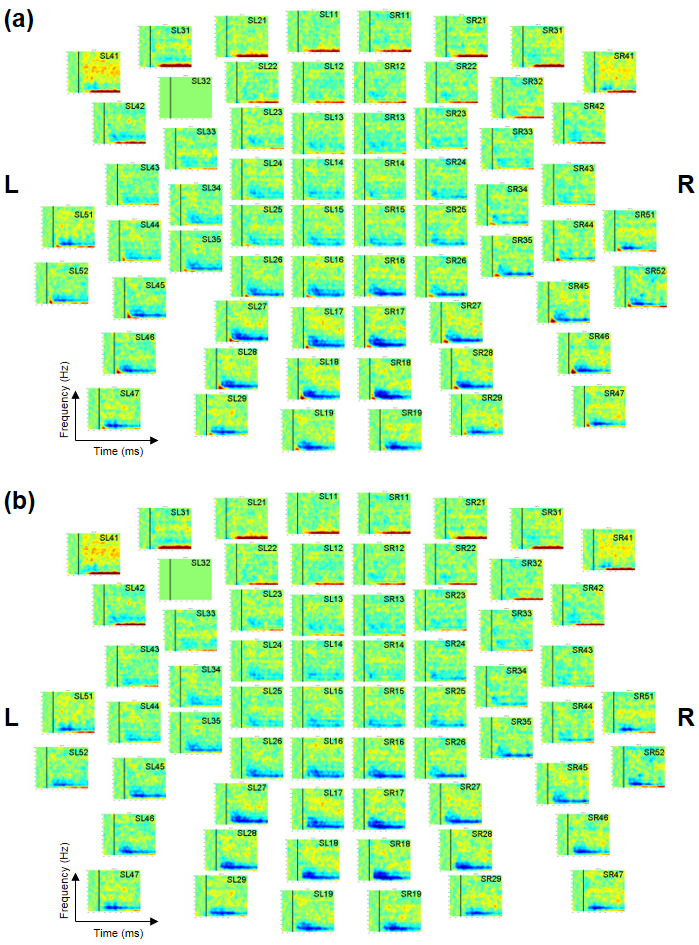
**

**Supplementary Figure 23.** Group average of sensor level result of time-frequency analysis of abacus experts during observation task. The vertical axis represents the frequency and the horizontal axis represents the time. For color-map, blue-red shows ERD and red colour shows ERS. (a) Spectrum of all sensors displaying both evoked responses and induced responses. (b) Spectrograms of all sensors showing only induced responses.

**
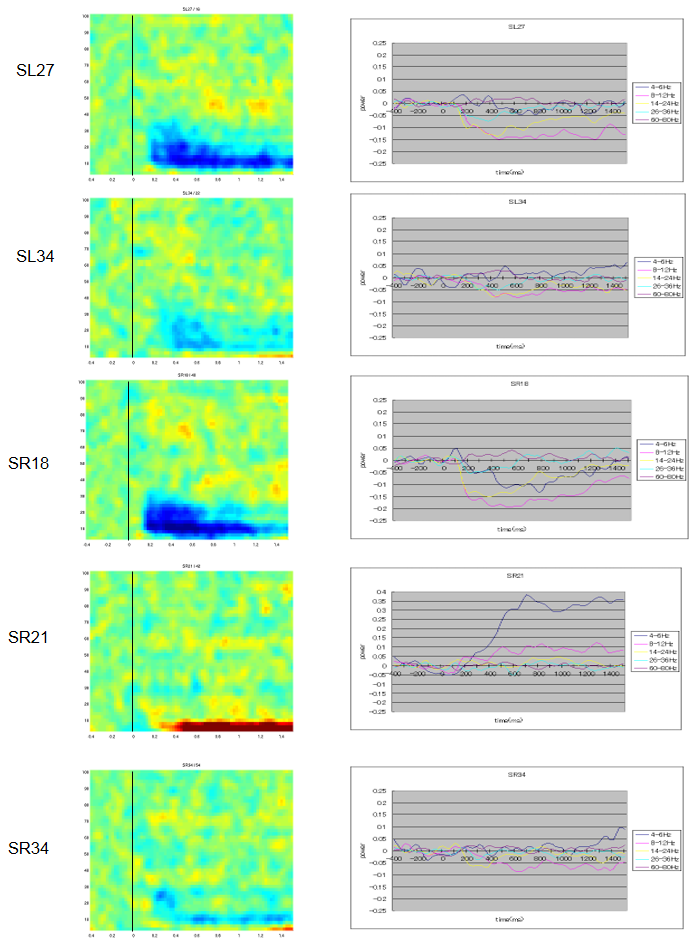
**

**Supplementary Figure 24.** MEG sensors with main brain activities (ERD and ERS) for abacus experts during observation task. For color-map, blue-red shows ERD and red colour shows ERS.


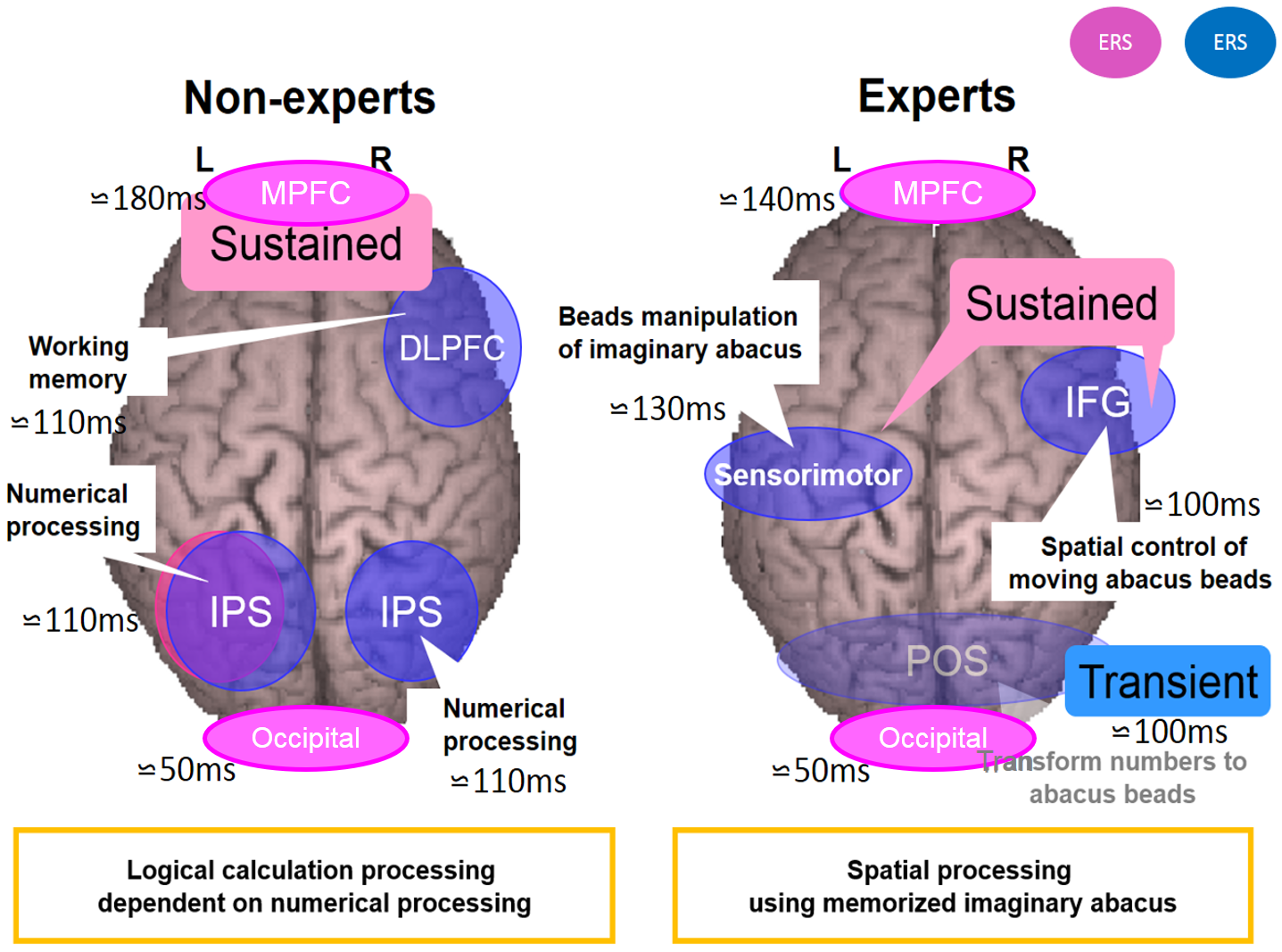


**Supplementary Figure 25.** The proposed calculation model of abacus and non-abacus experts taking into account of spatiotemporal profiles of oscillatory changes. We concluded that non-abacus experts use logical calculation processing dependent on numerical processing. While the abacus experts use spatial processing, using memorized imaginary abacus. For color-map, blue-red shows ERD and pink colour shows ERS.
